# Modeling Polyzwitterion-Based Drug Delivery Platforms: A Perspective of the Current State-of-the-Art and Beyond

**DOI:** 10.1021/acsengineeringau.2c00008

**Published:** 2022-05-03

**Authors:** Sousa Javan Nikkhah, Matthias Vandichel

**Affiliations:** † Department of Chemical Sciences, Bernal Institute, University of Limerick, Limerick V94 T9PX, Republic of Ireland

**Keywords:** Charged polymers, Polyzwitterions, Multiscale modeling, Molecular simulations, Drug delivery, Antifouling, Self-assembly, Intelligent drug delivery system

## Abstract

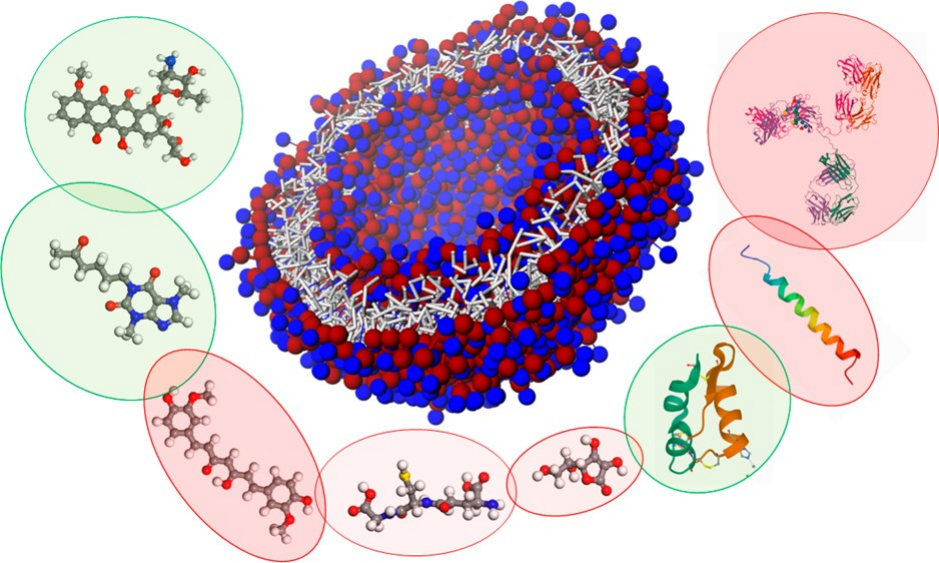

Drug delivery platforms are anticipated to have biocompatible and bioinert surfaces. PEGylation of drug carriers is the most approved method since it improves water solubility and colloid stability and decreases the drug vehicles’ interactions with blood components. Although this approach extends their biocompatibility, biorecognition mechanisms prevent them from biodistribution and thus efficient drug transfer. Recent studies have shown (poly)zwitterions to be alternatives for PEG with superior biocompatibility. (Poly)zwitterions are super hydrophilic, mainly stimuli-responsive, easy to functionalize and they display an extremely low protein adsorption and long biodistribution time. These unique characteristics make them already promising candidates as drug delivery carriers. Furthermore, since they have highly dense charged groups with opposite signs, (poly)zwitterions are intensely hydrated under physiological conditions. This exceptional hydration potential makes them ideal for the design of therapeutic vehicles with antifouling capability, *i.e*., preventing undesired sorption of biologics from the human body in the drug delivery vehicle. Therefore, (poly)zwitterionic materials have been broadly applied in stimuli-responsive “intelligent” drug delivery systems as well as tumor-targeting carriers because of their excellent biocompatibility, low cytotoxicity, insignificant immunogenicity, high stability, and long circulation time. To tailor (poly)zwitterionic drug vehicles, an interpretation of the structural and stimuli-responsive behavior of this type of polymer is essential. To this end, a direct study of molecular-level interactions, orientations, configurations, and physicochemical properties of (poly)zwitterions is required, which can be achieved via molecular modeling, which has become an influential tool for discovering new materials and understanding diverse material phenomena. As the essential bridge between science and engineering, molecular simulations enable the fundamental understanding of the encapsulation and release behavior of intelligent drug-loaded (poly)zwitterion nanoparticles and can help us to systematically design their next generations. When combined with experiments, modeling can make quantitative predictions. This perspective article aims to illustrate key recent developments in (poly)zwitterion-based drug delivery systems. We summarize how to use predictive multiscale molecular modeling techniques to successfully boost the development of intelligent multifunctional (poly)zwitterions-based systems.

## 1 Introduction

**Figure 1:**
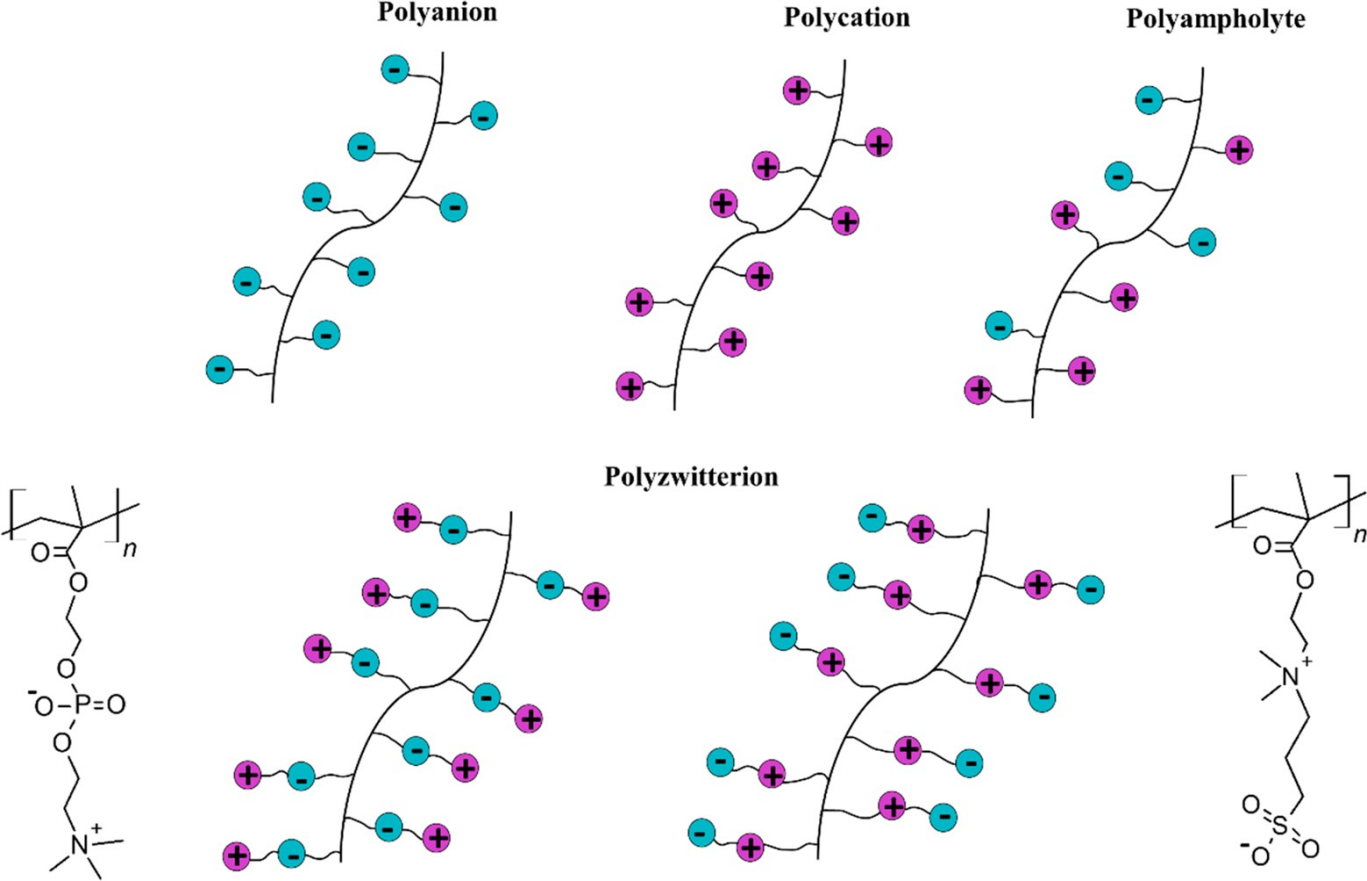
Schematic representation of polyanion, polycation, polyampholyte, and two types of polyzwitterion. The bottom-left and bottom-right panels show two examples of polyzwitterions: poly(phosphorylcholine) and poly(sulfobetaine), respectively.

**Figure 2:**
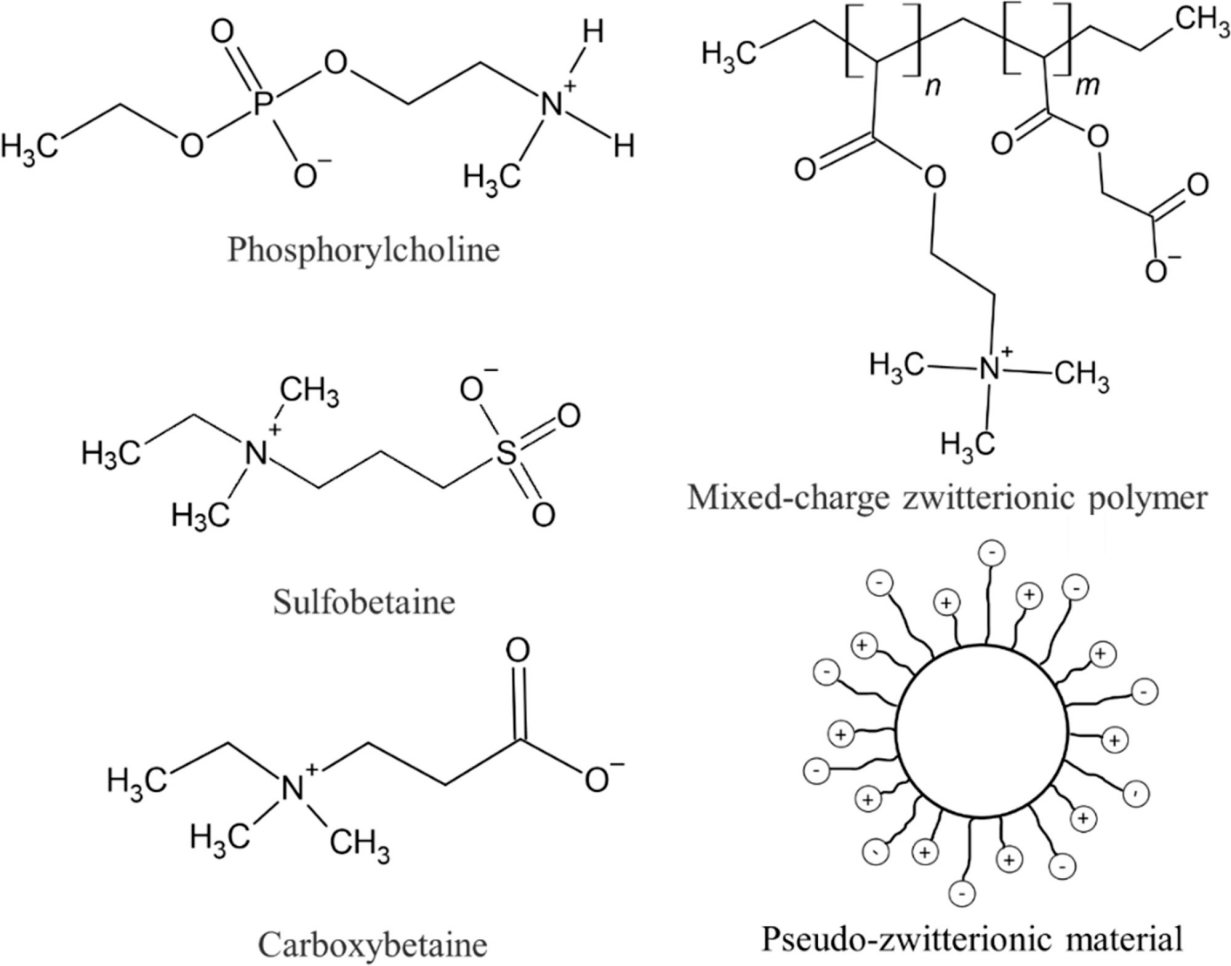
Chemical structures of the common zwitterionic groups, a mixed-charge zwitterionic polymer, and pseudozwitterionic materials with equimolar negative and positive charge binding to the same medium.

**Figure 3:**
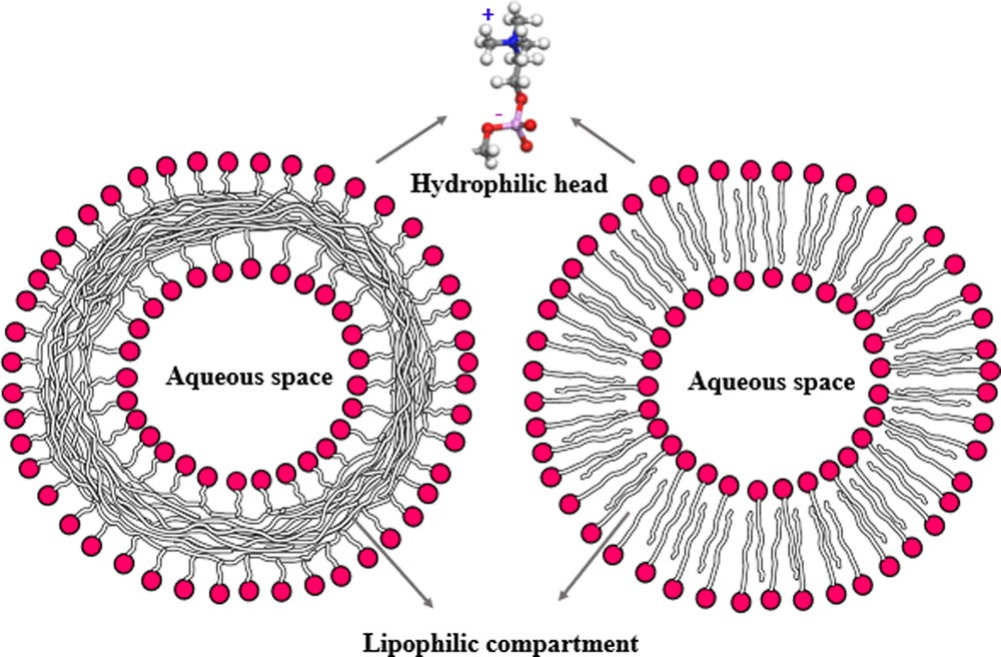
Schematic picture of a polymersome made from polyzwitterions (left panel) and a vesicle made from zwitterions (right panel). The amphiphilic (poly)zwitterions contain hydrophilic headgroups with both positive and negative charges and a hydrophobic tail or backbone, resulting in vesicles.

Polyzwitterions consist of oppositely charged cationic and anionic groups along the chain or side chain within their constitutional repeating unit (see Figure 1).^1,2^ Examples of such zwitterion repeating units are phosphorylcholine, sulfobetaine, carboxybetaine, zwitterionic amino acids/peptides, and other mix-charged zwitterions (see some examples in Figure 2). In the following, we use the term (poly)zwitterions referring to both zwitterions and polyzwitterions. During their self-assembly, they are both able to form a vesicle structure (Figure 3) and can thus both be used as platforms to encapsulate other molecules.^3−5^ Although (poly)zwitterions are characterized by highly dense polymer-bound ion pairs attached to the polymer backbone, their overall charge is zero under normal conditions. Thus, (poly)zwitterions illustrate a special subclass of polyampholytes presenting a very particular property profile.^6−9^ Polyampholytes act mainly as either polyanionic or polycationic species, whereas the overall charge neutrality of (poly)zwitterions makes them illustrate different hybrid-like properties (see Figure 1).^6^ In addition to their overall electroneutrality and high hydrophilicity, having large dipole moments and highly charged groups,^10^ polyzwitterions share numerous analogies with polar nonionic polymers.^6,11−13^ Unlike PEG, which binds water molecules via creating hydrogen bonds, the electrostatic ion–dipole interaction between zwitterions and water could form stronger, denser, and tighter hydration shells.^14^ This feature finally guides to ultralow nonspecific protein adsorption, bacterial adhesion, biofilm formation and makes zwitterions suitable substitutes for PEG.^5,15^

Although (poly)zwitterions are known at least from the late 1950s,^16,17^ they have been studied as rather peculiar materials for a while. (Poly)zwitterions can be manufactured into different structural forms: brushes,^18−20^ films,^21^ hydrogels,^22−24^ particles,^25^ membranes,^26^ and coatings,^27^ with different functions, *e.g.* antifouling,^23,24,27^ responding to stimuli,^28,29^ lubricating,^30,31^ self-healing,^32^ antibacterial,^24^ and biosensing.^33,34^ Due to their great tolerance to highly saline environments,^9^ they have remarkable potential to be applied as ionomers,^35,36^ fibers,^6^ and rheology modifiers,^37,38^ drug/gene delivery vehicles,^2,5,39,40^ analogues of important biological structures,^6,41^ and anti(bio)fouling materials.^15,42,43^ In addition to their outstanding antifouling property, zwitterionic materials can also enhance biocompatibility, reduce immune response, promote cellular uptake of chemical drugs/genes, and prolong the circulation time.^5,44^ Zwitterionic modifications could also provide various special functions when applied as drug carriers, such as stimuli-responsive and tumor targeting abilities.^5^ A summary of (poly)zwitterion platforms applied in drug delivery is presented in Table 1.

**Table 1:** Summary of Experimental (Poly)zwitterion Applications Related to Drug Delivery

name and type of (poly)zwitterion	loaded drug	description of specific properties	ref
poly(3-(3-methacrylamidopropyl-(dimethyl)-ammonio)propane-1-sulfonate) (PSPP)	DOX	pH-sensitive	(114)
copolymer (vinyl acetate (VA)-*co*-3-dimethyl(methacryloyloxyethyl)ammonium propanesulfonate (DMAPS))	potential drug delivery systems	effect of initial monomer feed on the copolymerization type, nanoparticles morphology, self-organization, and size distribution was studied	(115)
poly(VA-*co*-DMAPS)	nonsteroidal anti-inflammatory ibuprofen	*in vitro* water-insoluble, Ibuprofen release was studied experimentally to estimate the ability of microgels in improving the release process	(116)
poly(carboxybetaine) (PCB) grafted to branched polyethylenimine (PEI)	bovine serum albumin	PEI-PMPC-decorated nanoparticles showed better performance in tumor accumulation and anticancer ability than PEI–PCB-decorated nanopartciles	(117)
poly(2-methacryloyloxyethyl grafted to branched polyethylenimine (PEI) phosphorylcholine) (PMPC)			
polycation-*b*-polysulfobetaine block copolymer, poly[2-(dimethylamino) ethyl methacrylate]-*b*-poly[*N*-(3-(methacryloylamino) propyl)-*N*,*N*-dimethyl-*N*-(3-sulfopropyl) ammonium hydroxide] (PDMAEMA-*b*-PMPDSAH) luminescent carbon dots (CDs)	multifunctional gene delivery system	in the formed micelles, the CD cores acted as suitable multicolor cell imaging probes. In the formed micelle, the cationic PDMAEMA and zwitterionic PMPDSAH shell blocks acted as a DNA condensing agent and vector protecting against nonspecific interactions with serum components, respectively	(118)
star-shaped 3-dimethyl(methacryloyloxyethyl) ammonium propanesulfonate (DMAPS)		pH- and thermoresponsive	(119)
2-methacryloyloxyethyl phosphorylcholine (MPC)	therapeutic proteins uricase (uox) and l-asparaginase (l-asp)	MPC can be a candidate for enhancing the targeting efficiency in drugs, bioimaging, or biodetection delivery	(120)
poly(2-(*N*-oxide-*N*,*N*-diethylamino)ethyl methacrylate)	small-molecule anticancer drugs	adsorption of the polyzwitterion–drug conjugates to tumor endothelial cells and then to cancer cells helped their transcytosis-mediated extravasation into tumor interstitium and infiltration into tumors and led to the removal of large tumors and patient-derived tumor xenografts in mice	(121)
poly(carboxy betaine) (PCB)	l-asparaginase (ASP), a highly immunogenic enzyme drug	development of new therapies against diverse lymph-related diseases will be eased through this platform by enabling safe and efficient lymphatic drug delivery	(122)
polyzwitterion with acylsulfonamide-based betaine structure by one-step modification of polycarboxybetaine (PCB) with benzenesulfonamide	DOX	pH- and reductive-responsive	(123)
poly(sulfobetaine methacrylate)-*graft*-poly[oligo(ethylene glycol) methyl ether methacrylate)-*co*-di(ethylene glycol) methyl ether methacrylate] (PSBM-*g*-P(OEGMA-*co*-DEGMA)	potential drug delivery systems	thermoresponsive	(124)
zwitterionic polypeptides (ZIPPs) with a repetitive (VPX1X2G)_*n*_ motif, where X1 and X2 are cationic and anionic amino acids, respectively, and *n* is the number of repeats	glucagon-like peptide-1 (GLP1)	platform shows that a combination of lysine and glutamic acid in the ZIPP presents superior pharmacokinetics for intravenous and subcutaneous administration compared to uncharged control polypeptides	(125)
block copolymers of poly(ethylene glycol) (PEG) and sulfobetaine and sulfobetaine methacrylates		thermoresponsive	(126)
2-methacryloyloxyethyl phosphorylcholine-*b*-*n*-butyl methacrylate(MPC-*b*-PMB)	PTX	amphiphilic and biocompatible	(127)
2-methacryloyloxyethyl phosphorylcholine polymer bearing hydrazide groups(PMBH)	DOX, PTX	amphiphilic and pH-responsive	(128)
dithioester-capped 2-methacryloyloxyethyl phosphorylcholine-*co*-*n*-butyl methacrylate-*p*-nitrophenylcarbonyloxyethyl methacrylate (PMBN)	PTX	preS1 domain of hepatitis B targeting	(129)
poly-2-(methacryloyloxy)ethyl phosphorylcholine-*b*-poly-2-(diisopropylamino)-ethyl methacrylate (PMPC-*b*-PDPA)	DOX, PTX, dipyridamole	amphiphilic and pH-responsive	(130−133)
poly-2-(methacryloyloxy)ethyl phosphoryl-choline-*b*-poly 2-(diisopropylamino)ethyl methacrylate-*b*-poly[2-(dimethylamino)ethyl methacrylate] (PMPC-*b*-PDPA-*b*-PDMA)	DOX	pH-responsive	(134)
6-arm star poly(ε-caprolactone)-*b*-poly(2-methacryloyloxyethyl phosphorylcholine) (6sPCL-*b*-PMPC)	PTX	biodegradable	(135)
folic acid poly 2-(methacryloyloxy)ethyl phosphorylcholine-*b*-poly 2-(diisopropylamino)ethyl methacrylate] (FA-PMPC-*b*-PDPA)	tamoxifen, PTX	pH-responsive	(136)
poly-2-(methacryloyloxy)ethyl phosphorylcholine-*b*-poly(butyl methacrylate) (PMPC-*b*-PBMA)	PTX	amphiphilic	(137)
poly-2-(methacryloyloxy)ethyl phosphoryl-choline-*b*-poly(lactic acid) (PMPC-*b*-PLA)	DOX	biodegradable	(138,139)
poly(ethylene oxide) (PEO)-*b*-poly(2-methacryloyloxyethyl phosphorylcholine)/α-cyclodextrins (PEO-*b*-PMPC/α-CD)	DOX	amphiphilic and biocompatible	(140)
poly(β-amino ester)-*graft*-12-acryloyloxy dodecyl phosphorylcholine (PAE-*graft*-ADPC)	DOX	pH-responsive	(141)
cholesterol-end-capped poly(2-methacryloyloxyethyl phosphorylcholine) (CMPC)	DOX	amphiphilic and biocompatible	(142,143)
poly(lactic-*co*-glycolic acid)-*b*- poly(carboxybetaine) (PLGA-*b*-PCB)	docetaxel	galactose targeting	(144)
degradable shell cross-linked knedel-like nanoparticles composed of poly(acrylic acid)-based shells and poly(lactic acid) cores- poly(carboxybetaine) (dSCKs-PCB)		amphiphilic and biodegradable	(145)
poly(carboxybetaine methacrylate)-*co*-poly(2-(methacryloyloxy)ethyl lipoate)(poly(CBMA-*co*-MAEL))	DOX	amphiphilic and redox-responsive	(146)
1,2-distearoyl-*sn*-glycero-3-phosphoethanol-amine-*b*- poly(carboxybetaine) (DSPE-*b*-PCB)	DOX	amphiphilic and biocompatible	(147)
poly(carboxybetaine methacrylate)(PCBMA)	DOX	redox-responsive	(148,149)
1,2-distearoyl-*sn*-glycero-3-phosphoethanol-amine-*b*-polycarboxybetaine (DSPE-*b*-PCB)	insulin	platform for oral delivery of the protein that enables mucus penetration and thus efficient transporter-mediated epithelial absorption	(50)
poly(2-methacryloxyethyl phosphorylcholine-*co*- *n*-butyl methacrylate)	PTX	evaluation of the effect of injection of PTX prepared by solubilization with the amphiphilic copolymer of PMPC-*co*-PBMA, for peritoneal dissemination of gastric cancer	(150)

Among these diverse functionalities, the excellent antifouling behavior of (poly)zwitterions is still well recognized, which makes them comparable or even superior to PEG-based coatings.^45,46^ In recent years, (poly)zwitterions have attracted much attention in the field of biomaterials, mainly because of their outstanding properties.^47^ Due to their typical stimuli-responsive behavior (*e.g.*, temperature-, pH-responsive), they exhibit dual-nature properties, switching between anti-polyelectrolyte (zero net charge) or polyelectrolyte (non-zero net charge) behaviors depending on their environment; as such, they can be considered as “intelligent” or “smart” adaptive materials.^2^

If (poly)zwitterions have a strong amphiphilicity, they self-assemble in supramolecular structures.^48,49^ These amphiphiles contain headgroups that have both a positive and negative charge and hydrophobic tails (hydrophobic backbones for polyzwitterions), resulting in micelles,^50,51^ or lipid bilayer vesicles (see Figure 2).^52,53^ If, for instance, a (poly)zwitterion contains a carboxylate and a protonated ammonium ion, it may behave as a (poly)anion at high pH (due to deprotonation of the ammonium ions)) and it can act as a (poly)cation at low pH (due to protonation of the carboxylate groups)), assuming then an amphoteric character.^54^ This pH-responsive behavior has made (poly)zwitterions applicable in controlled drug release.^5,55^

To better understand the fundamentals of the behavior of (poly)zwitterionic molecules, especially in a self-assembly process, it is crucial to know how these outstanding molecules interact with themselves and biological molecules, more specifically their conformational and orientational properties in solution. Molecular recognition in charged polymeric systems, such as polyzwitterions and polyelectrolytes, relies on specific attractive and repulsive electrostatic interactions and attractive hydrophobic interactions (of the backbones).^56,57^

The rational design of drug delivery systems leveraging self-assembled supramolecular structures, such as surfactant/polymer micelles, (micro)emulsions, vesicle/liposomes, and layer-by-layer assemblies, has gathered notable interest in recent years in improving therapeutics.^58,59^ The undeniable importance of self-assembled supramolecular structures in transferring drugs originates from their capabilities to improve the physicochemical, pharmacokinetic, and pharmacodynamic characteristics of drugs to achieve efficient delivery of therapeutics to desired sites in the body.^60,61^ The supramolecular formation of (poly)zwitterions (*e.g*., micellization, vesiculation, and polymersome formation) is driven by electrostatic interactions; however, the entropy loss of polymer chains during this process also matters.^62,63^ Generally, the enthalpy–entropy compensation plays a crucial role in the self-assembly process of (poly)zwitterions. The enthalpy term favors the process mainly because of the water–water interactions. Water–water interactions, originated from the cohesive force, intensify by expelling (poly)zwitterions from the aqueous phase at the expense of weaker water–hydrophobic tail interactions. Another favorable term in the enthalpy is attributed to the electrostatic interactions between the positively and negatively charged fragments of the (poly)zwitterion. While the main entropy term that disfavors the process arises from ordering the (poly)zwitterion molecules by placing them in the bilayer phase that suppresses their freedom and thus causes entropy loss.^64−66^

It is needless to say how helpful having a deep understanding of the affinity contributions of attractive and repulsive interactions and the entropy contribution can be to engineer and tune supramolecular-based therapeutic vehicles. As one of the most effective tools, the multiscale modeling approach holds great promise in predicting the structure and response behavior of materials in various disciplines, including the design of drug carriers.^67,68^ Visualization of experimental data in a three-dimensional atomic-scale model can assist in explaining phenomena and often raises new questions, thereby improving future research.^69−73^ However, to study and design effective drug delivery systems using molecular modeling, a broad range of length and time scales needs to be spanned. In this regard, understanding the capabilities of different molecular modeling techniques such as *ab initio* quantum mechanics (QM), molecular dynamics (MD), Monte Carlo (MC) methods, and mesoscale (MS) methods in the drug delivery systems studies can assist us to select and well-parametrize proper modeling tools (more details of the simulation methods are presented in Table 2).^70,74−76^ From all these methods, QM techniques can explain any molecular systems behaviors at the atomistic level by computing the electron distribution but are limited in the system size they can tackle.^77^ All noncovalent interactions of various systems at atomic resolution can be monitored using MD simulations.^78,79^ However, many critical problems in the drug delivery field happen at larger time and length scales far beyond what can be tackled using atomistic MD (force field). Such problems can be modeled via mesoscale modeling techniques that have been adequately parametrized using atomistic simulations.^80^ Mesoscopic simulations are implemented using a coarse-grained molecular model that starts with a choice of the length scale for coarsening and then subsumes all atoms within selected specific length scales into one particle (bead).^74,81,82^ Some coarse-grained beads are connected by “bonds” (spring potentials) to reproduce an overall architecture of the reference atomistic model.^83^ Coarse-grained molecular dynamics (CGMD),^84,85^ MARTINI,^86,87^ and dissipative particle dynamics (DPD)^20,88−91^ are some of the most applied mesoscopic simulation approaches that have been employed to tackle ionic and nonionic polymeric materials, providing a deep understanding of design roles for the rational engineering of these novel drug carriers.^83^ Some selected examples of multiscale molecular modeling studies on (poly)zwitterions are presented in Table 2.

**Table 2:** General Description of the Typical Simulation Methods Applied to Study (Poly)zwitterionic Drug-Delivery Systems and Selected Examples

scales	length and time scales^151^	descriptions	examples of (poly)zwitterion modeling studies
quantum scale	∼10^–10^ m and ∼10^–12^ s	at the quantum scale, the nuclei and electrons are the main target^70^	effect of the distance between the oppositely charged groups, carbon spacer length, on molecular properties of zwitterionic carboxybetaines^152^
			investigation of the interactions between pirarubicin, an antibiotic, and zwitterionic distearoylphosphatidylcholine (DSPC) or anionic distearoylphosphatidylglycerol (DSPG)^153^
atomistic scale	∼10^–9^ m and 10^–9^–10^–6^ s	Monte Carlo (MC) method is a stochastic method that employs random numbers to generate and evaluate new configurations of the system. Ensemble integration allows then the calculation of the properties of interest^70,151,154^	coupled Monte Carlo (MC)/molecular dynamics (MD) approach to determine the interface potentials of water on polysulfone (PSF) based membrane^155^
			packing structure, surface hydration, and antifouling property of three zwitterionic polymer brushes of poly(carboxybetaine methacrylate) (pCBMA), poly(sulfobetaine methacrylate) (pSBMA), and poly((2-(methacryloyloxy)ethyl)phosporylcoline) (pMPC) were investigated^45^
		molecular dynamics (MD) simulation technique can predict the time evolution of a system of particles (*e.g.*, atoms, molecules, granules, *etc.*) interacting with each other and estimate the equilibrium, dynamics, and physical properties^70,151^	investigation of the structure and antifouling performance of the zwitterionic peptide brushes^156^
			interaction modes between glucagon-like peptide-1 and three types of zwitterionic pentapeptides^157^
			three single lipid component liposomes formed from the commonly used phospholipids: 1,2-dioleoyl-*sn*-glycero-3-phosphocholine (DOPC), 1,2-dipalmitoylphosphatidylcholine (DPPC), or phosphatidylcholine (POPC) were observed; the results indicate that the detachment of two leaflets of the DOPC surface is detached via ultrasound, suggesting that the drug release pathway may be through the low lipid packing areas on the stretched surface^158^
mesoscopic scale	∼10^–6^ m and 10^–6^–10^–3^ s	coarse-grained molecular dynamics (CGMD) methods overcome atomistic simulations’ length and time scale limitations through coarse-graining large molecules by several connected beads^70,83,86,159^	investigation of the interactions between AuNPs grafted with zwitterionic polymers and lipid membranes^160^
			two coarse-grained (POL- and BMW-MARTINI) models studied the interaction between a cationic gold nanoparticle functionalized with primary alkane amines and a lipid bilayer consisting of either zwitterionic lipids or a mixture of zwitterionic and anionic lipids^161^
		dissipative particle dynamics (DPD) technique is a mesoscopic simulation method that correctly accounts for hydrodynamic interactions; in DPD simulations, a cluster of atoms is represented by one bead, and Newton’s equation of motion governs its dynamics^70,151,162−164^	stability and drug loading/release behavior of unimolecular micelles formed using generation-5 polyamidoamine-*graft*-poly(carboxybetaine methacrylate) (PAMAM(G5)-PCBMA) was investigated^165^
			poly(ε-caprolactone)-*b*-poly(diethylaminoethyl ethacrylate)-*b* poly(sulfobetaine methacrylate), a pH-sensitive amphiphilic triblock copolymer was studied as a carrier for doxorubicin (DOX)^166^

The field of (poly)zwitterions has occasionally been reviewed, in which the majority of reviews focus on only one specific aspect, such as synthetic polyzwitterion applications,^92^ phospholipids polymers,^93,94^ polyzwitterionic membranes,^95^ zwitterionic gel,^96^ antifouling behavior of (poly)zwitterions,^42,97−99^ (poly)zwitterion/biological species interface,^100^ synthesis, and structures of (poly)zwitterions,^6,8,12,101−103^ surface properties of polyzwitterions,^2,98,104^ (poly)zwitterion coatings,^105^ and stimuli-responsive behavior of polyzwitterions.^18,106^ In this Perspective, we summarize the recent studies of (poly)zwitterion applications in drug delivery systems using multiscale molecular modeling techniques. We focus on the features of these materials and discuss the drug delivery applications from three different angles:

(1) **Self-assembly**: We discuss amphiphilic zwitterionic materials that can self-assemble and form smart drug carriers, such as stimuli-responsive micelles and liposomes or vesicles. More specifically, vesicle morphologies are important in view of biological membranes. Since their structure mimics a cell membrane, vesicles are membrane models for physical and chemical studies; *e.g*. drug carriers to solubilize and incorporate biomolecules and to target cells.^107^
(2) **Surface modification**: Although drug delivery nanoparticles have attracted significant interest in diagnosing and treating many human diseases, their cytotoxicity and uptake efficiency significantly limit their clinical use.^108^ Fortunately, it is possible to tune their physicochemical properties to boost their biocompatibility and uptake efficiency through the functionalization of the surface of the nanoparticles.^109^ In this regard, surface modification by zwitterionic materials allows tuning of charge densities to improve the solubility and increase their stability in biological fluids. This modification increases targeted uptake and their accumulation in target tissues.^110,111^
(3) **Membrane**: The zwitterionic lipid bilayer is another topic of interest since such bilayers mimic the biological cell membranes and control the flow of materials in and out of the cell.^112,113^ The importance of modeling this type of membrane originates from the fact that transport of drugs (or drug delivery systems) over the cell membrane is a complicated biological process. In this regard, the model of zwitterionic lipid membranes is very useful in assisting researchers in perceiving the roles of lipid membranes in cellular interactions. The usual drug properties required to be predicted in this type of modeling study can be pharmacokinetic properties of drugs such as their distribution, accumulation, and transport mechanism by screening drug-membrane interactions and drug orientation in the system.

**Figure 4:**
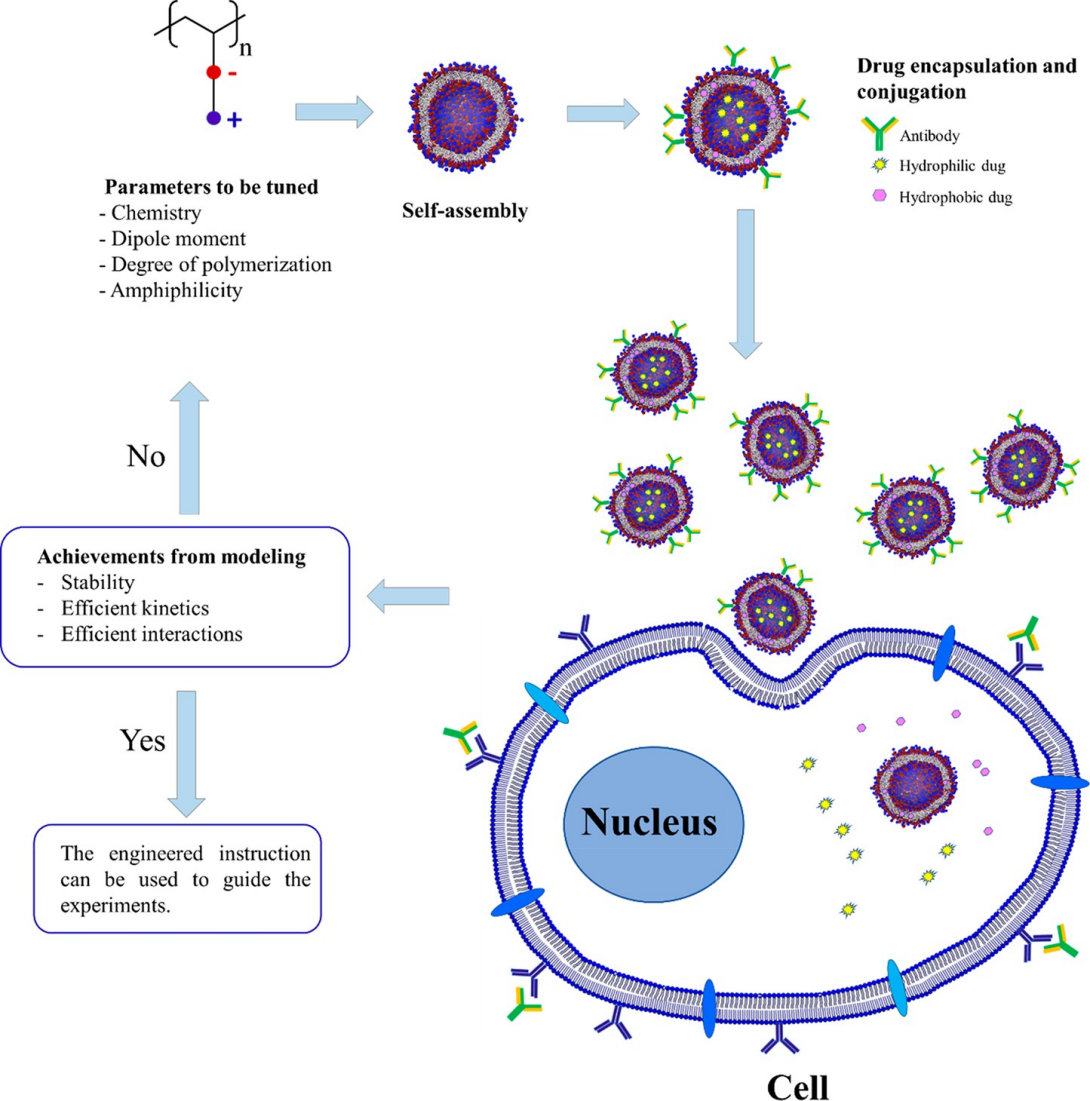
Graphical representation of the algorithm to be followed for engineering (poly)zwitterionic-based intelligent drug delivery systems via molecular modeling.

The use of zwitterion-based drug delivery particles to carry drugs to their target cells is one of the advantages of incorporating intelligent stimuli responsive materials within medicine. Notably, their ability to functionalize surfaces and respond to stimuli and their antifouling behavior enable them to properly encapsulate and protect drugs and target cells more effectively. Understanding how these carriers enter cells is critical for deciphering the intercellular dose provided for the target cells to determine the efficacy of utilizing zwitterion-based intelligent drug delivery and advancing drug delivery systems. In this context, two main issues need to be focused: (1) Designing the drug delivery platform and (2) tracking the platform in crossing cells and releasing the drug. We have chosen the above subsections because we believe that, through subsections 1 and 2, design and advancing the drug delivery systems, and in section 3, their ability to cross the cells and cellular uptake efficacy can be surveyed. The graphics of the corresponding algorithm with the above-mentioned issues (and the subsections) to be followed to engineer desired (poly)zwitterion-based drug delivery systems is presented in Figure 4. We hope our Perspective will encourage the readers to conduct more modeling-guided explorations of (poly)zwitterion-based templates as intelligent drug delivery systems. The motivation of this work is to highlight how multiscale molecular modeling techniques can assist us in designing such intelligent (poly)zwitterionic based therapeutic delivery vehicles, nanocarrier surface engineering using (poly)zwitterions, membranes formation, and monitoring drug penetration mechanism through zwitterion bilayer membranes.

## 2 Zwitterions and Polyzwitterions

Let us briefly describe the (poly)zwitterions nature, characteristics, potential, and typical applications. Zwitterions are small molecules that include spatially separated positive and negative charges (see Figures 1 and 2). Generally, the formats of (poly)zwitterions contain monolayers, bi- or multilayers, layer-by-layer films, polymer brushes, and polymer networks.^167,168^ Two prominent examples of zwitterions in nature are phospholipid bilayers in cell membranes and special osmolytes in plants or animals.^103,167,169^ However, bioinspired synthetic polymers with zwitterionic groups in their repeating units (*i.e.*, polyzwitterions) have emerged as an affluent area of study with a main focus on artificial membranes, drug delivery, water treatment, and biomedicine.^92,167^

Zwitterionic materials can be generally classified as betaine-like zwitterions and mixed-charge zwitterionic materials depending on whether the cationic and anionic groups are on the exact same unit of zwitterions (see Figure 2). Most of the zwitterions belong to the betaine group and these always contain quaternary ammonium as cations. Typical examples of betaines are phosphorylcholine (PC), sulfobetaine (SB), and carboxybetaine (CB), with as anion groups phosphonates (PO_3_^–^), sulfonates (SO_3_^–^), and carboxylates (COO^–^), respectively. However, there are some mixed-charge materials, including positively and negatively charged moieties in different monomer units, to maintain the overall electrical neutrality.^170^ These mixed-charge zwitterionic materials show similar antifouling properties because of their resembling structures.^171,172^ Furthermore, amino acids and peptides can be considered natural zwitterions, such as serine, ornithine, lysine, aspartic acid, and glutamic acid, which also have presented considerable anti(bio)fouling and better biocompatibility than many synthetic (poly)zwitterionic polymers.^5,173,174^

As hydrophilic materials, zwitterions have strong intra- and intermolecular ion-pairs.^15^ Consequently, at not so low pHs that the anionic groups do not get protonated, zwitterions have a zero effective net charge. However, if the nitrogen is not quaternary, there will be an upper limit of pH beyond which the zwitterionic character will also be lost.^6,103^

Their high density of charged groups with opposite signs, on the other hand, makes them strongly hydrated under physiological conditions. For this reason, bioadhesion (adhesion of biological matters, such as proteins) to (poly)zwitterions is strongly reduced. Their biocompatibility is another valuable aspect that has made them valuable for medical applications, which arises from their nontoxic behavior, ability to sustain the healthy functions of surrounding tissue, noninflammatory causing behavior, and being tissue-integrated without encapsulation. Thus, (poly)zwitterions, as protein-repellent, nonthrombogenic, and cell-compatible materials, are attracting scientific interest, particularly in the field of biomedicine.^101^

In the following, we assess recent modeling-led research that studied (poly)zwitterionic drug delivery systems. We aim to amplify the importance of modeling-based research in the characterization of (poly)zwitterionic materials, the design of self-assembled zwitterionic-based supermolecular structures, and their surface engineering for applications in drug delivery. The current state-of-the-art of these two application platforms forms the focus of the following subsections.

### 2.1 Self-Assembly of (Poly)zwitterions

**Figure 5:**
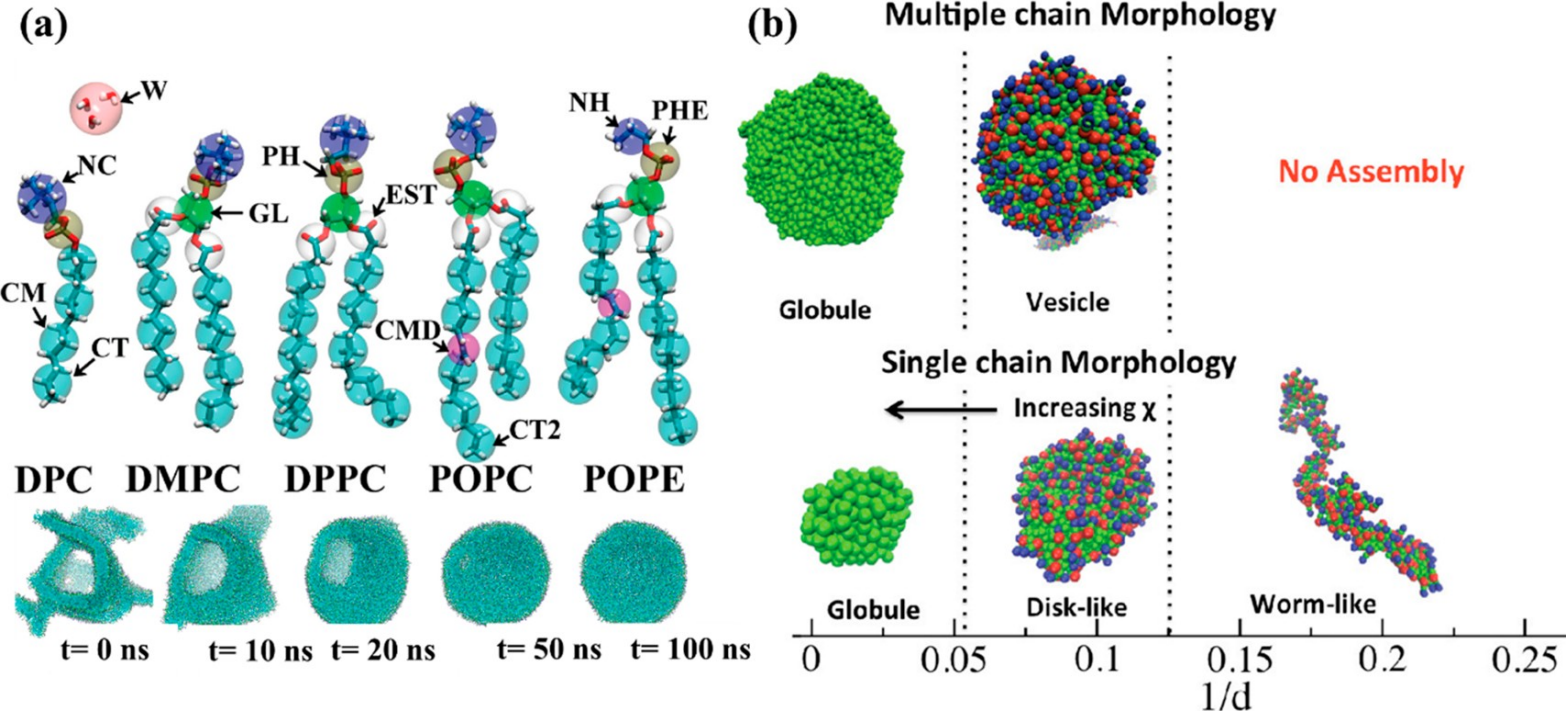
(a) Left top panel: Schematic definition of coarse-grained sites, left bottom panel: Transformation of self-assembled lipid aggregates in a large water box. The five rows represent lipid aggregates of 5000 DMPC molecules, respectively. Adapted from ref (185). Copyright 2010 American Chemical Society. (b) Single chain structures and multiple chain morphologies as a function of 1/*d*. The single chain transitions from globule → disk → worm-like structures with increase in 1/*d*. The polymer chains aggregate to form bigger globules for 1/*d* = 0.000 (45 single chains), bowls, and/or vesicles (28 single chains) for 0.000 < 1/*d* < 0.125, and they do not aggregate for 1/*d* ≥ 0.125 (*N* = 100). χ represents the Flory–Huggins interaction parameter. Adapted with permission from ref (4). Copyright 2016 American Institute of Physics.

(Poly)zwitterions display various solubility properties and self-assembled morphologies in an aqueous solution.^175−178^ The formation of vesicles by some polyzwitterion architectures,^179^ is one of the particular interests due to the vesicles’ potential applications in drug delivery. It is generally known that micelles^50^ and vesicle-like morphologies such as polymersomes (see Figure 3) form easily by polyamphiphiles,^180−184^ through a self-assembly process in which hydrophilic and hydrophobic interactions are optimized. However, the unique properties of polyzwitterion assembly is the involvement of the long-ranged dipolar forces emerging from the zwitterion groups attached to the hydrophobic polymer backbone. Different researchers have studied the self-assembly of (poly)zwitterions using molecular modeling, employing the coarse-graining method. In the following, we review some modeling-led research implemented to study the self-assembly process of (poly)zwitterions. Shinoda et al.^185^ presented a coarse-grained (CG) intermolecular force field to model a series of zwitterionic lipids; their study includes a dodecylphosphatidylcholine (DPC) micelle solution and four different bilayers: dimyristoylphosphatidylcholine (DMPC), dipalmitoylphosphatidylcholine (DPPC), palmitoyl oleyl phosphatidylcholine (POPC), and palmitoyl oleyl phosphatidylethanolamine (POPE) (see Figure 5a). For their model, they optimized the force field parameters for multiple lipid molecules using simple functional forms. The resulted CG lipid bilayers exhibited reasonable molecular areas, chain order parameters, and bilayer elastic properties. Since the persistence length of the DPPC monolayer is longer than that of a polyethylene glycol (PEG) lipid monolayer, a stable curved monolayer surface under negative tension was perceived for the zwitterionic monolayer. They also successfully observed vesicle formation in the system including DMPC molecules. Furthermore, their results revealed that, depending on the aggregate size, the lipid assembly spontaneously transforms into a closed vesicle or a bicelle (see Figure 5a). They also showed that the proposed CG force field could also support stable multilamellar DMPC vesicles.^185^ Due to the nanometer size of the simulated vesicles in this study and difficulties in performing a lab experiment for the self-assembly process, the study’s findings can shed light on the fundamental molecular level aspects of zwitterionic-based vesicle fusion, formation, and stability beyond the nanoscale. The importance of the obtained atomistic level perspectives is beyond only understanding the zwitterionic-based vesicle formation mechanism, but it can be generalized to one of the crucial phenomena in biological and nanotechnology applications, *i.e*., lipid assembly. In particular, in nanotechnology-led drug delivery, lipid assembly can lead to one of the demanding intelligent drug delivery platforms, i.e. liposomes (vesicles). Liposomes are appealing biomimetic nanocarriers widely used to carry different categories of therapeutics, such as hydrophobic and hydrophilic drugs, peptides, proteins, and antibodies.^186,187^ The structural versatility of liposomes has been utilized to develop numerous carriers for the systemic delivery of drugs, with the possibility of improving their bioavailability and stability and conducting their release while limiting the side effects simultaneously.^186^ This modeling-based study elaborates on the potential abilities of coarse-grain modeling to capture an insightful understanding of liposome (vesicle) formation at the nanometer level (see the time and length scales in Table 2), which provides knowledge to design novel liposomal drug vehicles more intelligently. Undoubtedly, due to the importance of liposomes in intelligent drug delivery systems, this type of modeling-based study of zwitterions self-assembly can be a starting point for a long road of predictive modeling studies to rationally design liposomal zwitterion-based drug delivery. However, there are challenges in investigating realistic vesicle formation processes, such as (1) a need for systematic CG force field parametrization that can be aided by developing density functional calculations and (or) MD modeling besides available experimental data; (2) and due to the loss of degrees of freedom that occurs through the mapping between an atomistic representation and a coarser one, specific physical interactions between system components are no longer present. Thus, even though careful parametrization may compensate for those missing interactions, special care needs to be taken to interpret CG simulation results, particularly when establishing the systematic errors associated with the results. Applying the Langevin dynamics method and a coarse-grained model, Mahalik and Muthukumar^4^ studied vesicles formation from hydrophobic polymers including zwitterions as side groups in dilute salt-free aqueous solutions. In their model, the zwitterions, as permanent charged dipoles, provide long-range electrostatic interactions that were influenced by the polymer’s conformational entropy. This competition between hydrophobic interactions and dipole–dipole interactions led to a series of self-assembled structures. Their results revealed that by decreasing the spacing between the successive zwitterion side groups, *i.e.*, *d*, single chains experienced globule → disk → worm-like structures (see Figure 5b).^4^ Since vesicle size can be from 100 nm to 1 μm, coarse-graining modeling is the suitable technique to capture the morphology evolution (see Table 2). They also monitored the effect of *d* on the vesicle structure, such as the radius of gyration, hydrodynamic radius, spatial correlations between hydrophobic and dipole monomers, and dipole–dipole orientational correlation functions. According to their results, during the self-assembly, these structures formed larger globules and vesicles by decreasing ‘d’ up to a threshold value, below which no large assembly formed. Similar to zwitterions, there is a particular interest in forming vesicles (polymersomes) by some polyzwitterion architectures given potential applications in drug delivery. These simulation results are likely to motivate theoretical work on assembly mechanisms for the less known field in dipole-bearing polymers; polyzwitterions assembly. In polyzwitterion self-assembly, there are two main aspects: (1) hydrophobic interactions of the backbones and (2) electrostatic interactions of the charged segments, both of which make their self-assembly process more complicated than zwitterions. A coarse-graining study can predict the effect of different system parameters, such as molecular weight, polyzwitterion architecture, and dipole moment that can control the self-assembly process. Through CG studies, different assembled morphologies will be achieved which can have potential applications as novel drug delivery platforms.

Liao *et al.*(188) performed DPD simulations to study the self-assembled morphologies of two copolymer systems containing PEG and the polyzwitterion, poly(carboxybetaine) (PCB), in aqueous solutions. The effects of the polymer composition and concentration on the self-assembled morphologies of the two amphiphilic copolymers were studied; one contains a hydrophilic block of PEG and another a block of PCB, while both contain a hydrophobic block of poly(lactic acid) (PLA) (PLA-*b*-PEG and PLA-*b*-PCB). Their results revealed that, regardless of the copolymer composition, PLA-*b*-PEG systems self-assembled into core–shell structures, whereas in addition to core–shell morphology onion-like and vesicle structures were also found for the PLA-*b*-PCB systems. For both copolymer systems, the final morphology was dependent on the polymer concentration. It is worth noting that, among different coarse-graining techniques, DPD can tackle the largest mesoscale systems due to the simple term of the conservative force^189^ and largest time and length scales (see Table 2). The simulation results also demonstrated that, at the same polymer concentration, the PLA-*b*-PEG self-assembled into a dumbbell-like structure while PLA-*b*-PCB formed a spherical one, showing the higher stability of PCB in maintaining self-assembled spherical structures than PEG. Although both copolymer systems could self-assemble into core–shell nanoparticles, the PEG shell layers formed in PLA-*b*-PEG nanoparticles were inhomogeneous due to the amphiphilicity of PEG, whereas the PCB shell layers in PLA-*b*-PCB nanoparticles were homogeneous because of the strong hydrophilic nature of PCB block. The work of Liao *et al.* is expected to provide valuable insight into the microscopic origins of the structural differences between the PEG-based and polyzwitterion-based drug delivery systems for the further development of these types of drug vehicles. Due to the simple format of the non-bonded potential term in DPD modeling, the time and length scales of mesoscale DPD modeling simulations can go beyond what is possible with other coarse-grained modeling techniques. This provides the opportunity to implement larger simulations, even comparable to the experimental size and in particular for (poly)zwitterions with high degrees of polymerization.

#### 2.1.1 Drug/Gene Delivery

**Figure 6:**
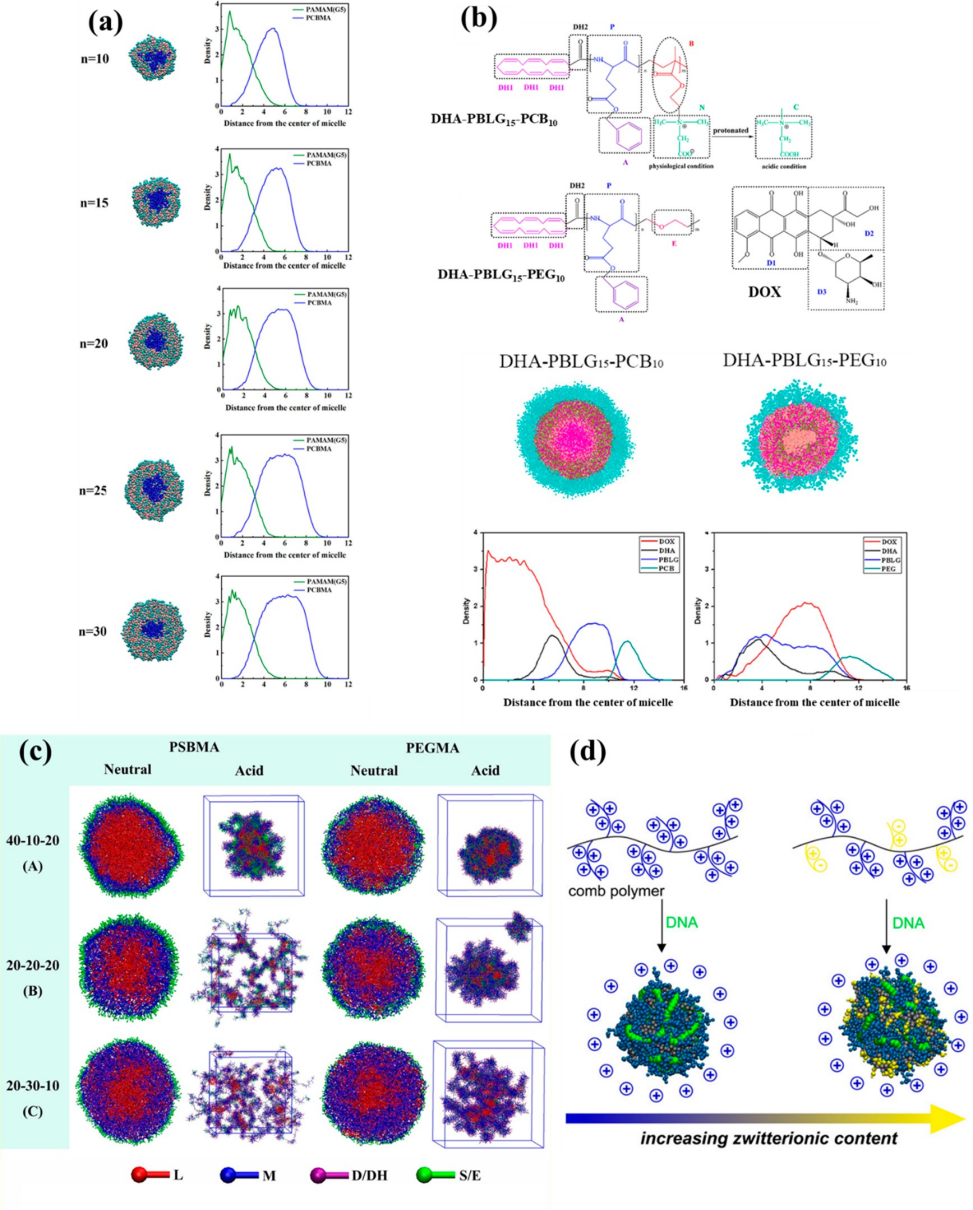
(a) Self-assembly morphologies of PAMAM(G5)-PCBMA unimolecular micelles at different PCBMA polymerization degrees: (left) sectional views and (right) density profiles of different segments. Adapted from ref (165). Copyright 2021 American Chemical Society. (b) Coarse-grained models of DHA-PBLG_*n*_-PCB_*m*_ and DHA-PBLG_*n*_-PEG_*m*_, (top panel) and^197^ Comparison of self-assembly morphologies of DOX-loaded copolymer DHA-PBLG_15_-PCB_10_ and DHA-PBLG_15_-PEG_10_ sectional views and density profiles of different beads (bottom panel). Adapted from ref (197). Copyright 2019 American Chemical Society. (c) Configurations of the blank micelles at different block lengths and different pH values in an aqueous solution. Water beads are eliminated for clarity (the same below). Adapted with permission from ref (166). Copyright 2017 Elsevier. (d) Dispersing zwitterions into comb polymers on the polyplex structure. Adapted from ref (200). Copyright 2016 American Chemical Society.

Herein, we discuss some research attempts to study self-assembled supramolecular (poly)zwitterionic structures for use in drug transferring with the aid of molecular modeling. Zeng *et al.*(165) studied the stability and drug loading/release mechanisms of unimolecular micelles formed using a zwitterionic dendrimer, generation-5 polyamidoamine-*graft*-poly(carboxybetaine methacrylate) (PAMAM(G5)-PCBMA), via coarse-grained DPD simulations. First, let us briefly mention the features and potential of zwitterionic dendrimers in drug delivery. Dendrimers have become ideal candidates as carriers in biomedical applications due to having highly controllable architectures, internal cavities, and the possibility for multivalent functionalization.^190^ However, the most commonly used dendrimers, such as PAMAM, by themselves are not biocompatible and can induce cytotoxic effects.^191^ Therefore, to reduce their toxicity for *in vivo* applications, the exterior of dendrimers is modified with, for example, PEG or charged groups.^192−194^ That is how polyelectrolyte dendrimers and zwitterionic dendrimers have emerged in designing drug vehicles.^195,196^ According to the simulations PAMAM(G5)-PCBMA spontaneously forms core–shell unimolecular micelles. The studied PAMAM(G5) dendrimer constituted a hydrophobic core to load the DOX, while the zwitterionic PCBMA acts as a protective shell to improve the stability of the unimolecular micelle (see Figure 6a). The results revealed that the DOX could be loaded into a PAMAM(G5) cavity at the physiological pH of 7.4. Regarding the release process, they observed that, at the acidic pH of 5.0, the loaded DOX could be released gradually from the hydrophobic core (see Figure 6a). This study demonstrates the potential of the zwitterionic dendrimer unimolecular micelles in drug transport from the molecular level that can offer theoretical guidance for designing and developing promising unimolecular drug delivery micelles. Let us outline the important issues in this research from scientific to technical points of view as a roadmap for future research: (1) If a drug delivery platform with high stability is required, unimolecular micelles can be one of the ideal options, (2) zwitterionic dendrimers can be applied as a stable intelligent drug vehicle taking advantage of the unique architecture, features of dendrimers, biocompatibility, and antifouling behavior of zwitterions, and (3) the DPD modeling technique can be a suitable choice to study zwitterionic dendrimer-based drug delivery systems, due to the large time and length scales that need to be covered (see Table 2) and the possibility of force field parametrization using Flory–Huggins solution theory.

Hao *et al.*(197) investigated the self-assembled behaviors of the zwitterionic copolymer docosahexaenoic acid–*b*-poly(γ-benzyl-l-glutamate)–*b*-poly(carboxybetaine methacrylate) (DHA-PBLG-PCB) and the loading and release mechanism of the anticancer drug DOX via DPD simulations (see Figure 6b for coarse-graining details). They explored the effects of polymer concentration, drug content, and pH on zwitterionic copolymer self-assembly. Simulation results showed that DHA-PBLG15-PCB10 self-assembled into pH-responsive core–shell micelles. According to their results, DOX could be encapsulated into the core–shell micelle under normal physiological pH conditions, while DOX release occurred under acidic pH conditions. The behaviors of the self-assembled copolymer DHA-PBLG-PEG were also studied and compared with those of DHA-PBLG-PCB. The results showed that the DHA-PBLG15-PCB10-based micelles were more stable than those of DHA-PBLG-PEG. This renders DHA-PBLG-PCB a great potential to serve as a drug vehicle for targeted delivery (see Figure 6b).^197^ This study is, in fact, a good example that indicates, with the help of DPD simulation and theoretical analyses, researchers could be guided in the design, preparation, and optimization of drug carriers based on linear triblock zwitterionic copolymer. It is worth noting that the applied approach in this study can be generalized to study linear multiblock zwitterionic copolymers, particularly with stimuli-responsive behavior.

Min *et al.*(166) performed DPD simulations to study the self-assembled microstructures and DOX loading/release properties of pH-sensitive amphiphilic triblock copolymers: poly(ε-caprolactone)-*b*-poly(diethylaminoethyl methacrylate)-*b*-poly(sulfobetaine methacrylate) or poly(ethylene glycol methacrylate) (PCL-PDEA-PSBMA/PEGMA). According to their results, both copolymers could successfully self-assemble into core–shell-corona micelles in an aqueous environment (see Figure 6c). However, the structures of the micelles’ corona were entirely different. The shell layers formed by PEGMA had a heterogeneous structure, while the shell layers in PCL-PDEA-PSBMA micelles were homogeneous. This was mainly attributed to the stronger hydrophilicity of PSBMA compared to PEGMA. They also observed that by increasing the mole fraction of copolymer from 10% to 50%, the microstructures formed by PCL-PDEA-PSBMA and DOX remained spherical micelles, whereas PCL-PDEA-PEGMA experienced a structural transition from spherical to cylindrical and finally to lamellar micelles (see Figure 6c). They also revealed that the drug release process followed a “swelling–demicellization–release” mode. This multiscale modeling study demonstrates an avenue to design nanomaterials for drug delivery and optimize their properties.^166^ Such a computational coarse-graining approach can be suitable to provide a deep understanding of existing experiments on systems of interest by monitoring them at both microscopic and mesoscopic levels, which causes raising the efficacy of experiments. Furthermore, it might provide some useful guidelines for optimizing and designing future novel biomolecules for intelligent drug delivery with desired properties, for instance, nonlinear multiblock zwitterionic copolymers.

Gene delivery systems based on polymeric materials benefit from the presence of hydrophilic groups that provide tunable polymer–DNA binding strength and stable polyplexes.^198−200^ However, hydrophilic groups screen charge, which could reduce cellular uptake and transfection efficiency. Using a combination of experiments and CG-MD simulations, Ghobadi *et al.*(200) studied the effect of attaching zwitterionic sulfobetaine (SB) groups to cationic comb polymers. In the beginning, they synthesized comb polymers with tetralysine (K4) and SB pendant groups through ring-opening metathesis polymerization (ROMP). They could successfully describe the effect of SB groups on the structure of comb polymer–DNA polyplexes, such as the shape, size, composition, surface charge, and polyplexes-DNA binding strength through both CG-MD simulations and experimental measurements. The simulation and experimental results concluded that increasing SB composition in the comb polymers caused a decrease in polymer–DNA binding strength. The CG-MD simulations specifically revealed that SB groups were distributed throughout the polyplex (see Figure 6d). This SB distribution is helpful to provide high levels of gene expression in living cells due to the positive charge of polyplexes surfaces. They also showed that the positive surface charge of the formed polyplexes from comb polymers, with nearly 50 mol.-% SB was similar to the polyplexes formed from purely cationic comb polymers, indicating the ability to add an substantial amount of SB functionality without screening the surface charge. This integrated computational-experimental study demonstrates the effectiveness of incorporating zwitterions in polyplexes, to design new and effective gene delivery vectors. The applied methodology in this study can be generalized as a suitable tool to engineer novel zwitterionic materials for the application in peptide, protein, antibody, gene and DNA carriers.

### 2.2 Surface Engineering with (Poly)zwitterionic Materials

Nanoparticle drug-delivery tools, such as liposomes,^201^ micelles,^202^ dendrimers,^203^ hydrogels,^204^ and virus-like nanoparticles,^205^ have emerged for different therapeutic applications to improve the specificity of drug actions and reduce the systemic side effects.^206^ However, their massive interactions with the surrounding physiological environments cause their rapid elimination from the blood circulation by the body’s immune system and thus drug release at off-target sites.^207,208^ By grafting a stealth coating layer onto the surface of nanoparticle drug carriers, the blood circulation half-life of nanomaterials can be improved. PEG and (poly)zwitterion are two typical polymers used for stealth coating.^209−211^ Compared to PEG coatings, polyzwitterion coatings, such as poly(sulfobetaine), poly(carboxybetaine), and poly(phosphorylcholine), could be better candidates as the oppositely charged groups within a polyzwitterion chain bind water molecules stronger than PEG chain molecules,^97^ and polyzwitterion coatings are chemically more stable than PEG coatings.^212^ In this regard, polyzwitterions are now used in marine coatings, disease diagnostics, and medical and biomedical applications.^15,213−216^

**Figure 7:**
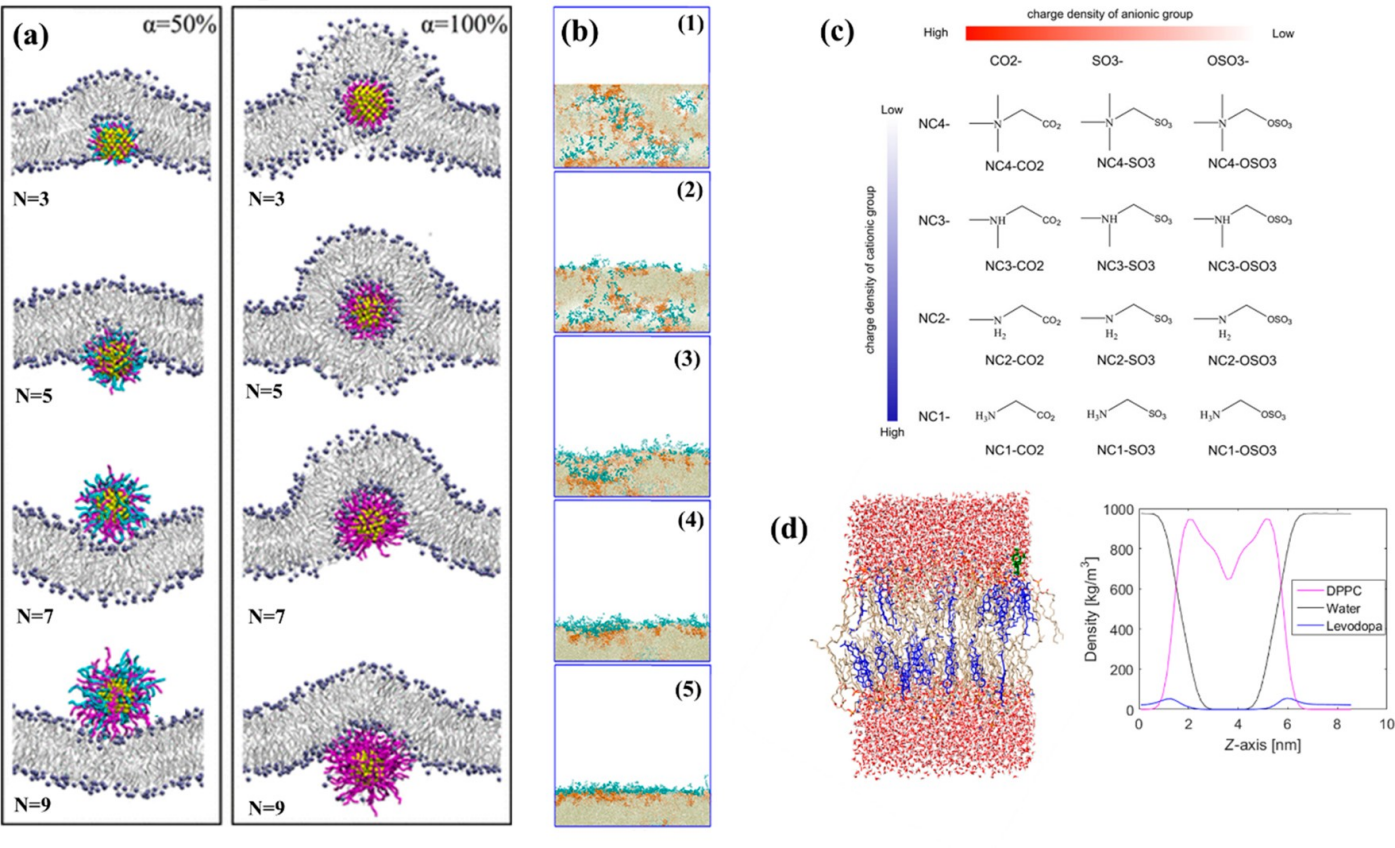
(a) Final equilibrated configurations of zwitterionic-AuNPs with different polymer chain lengths interacting with lipid membranes at (left) 50% and (right) 100% protonation degree, respectively. Water molecules are not displayed for clarity. The lipid headgroups are shown in blue, lipid tails in silver, gold core in yellow, PEG in green, the zwitterionic polymer in blue, and zwitterionic polymer after protonation in magenta. Adapted from ref (160). Copyright 2017 American Chemical Society. (b) PES-*b*-PCBMA/PES membrane (the blend of PES-*b*-PCBMA copolymer and PES homopolymer) formation process via nonsolvent induced phase separation (NIPS). Depicted are the initial state at (1) step 0; (2) step 20 000; (3) step 100 000; (4) step 20  000; (5) step 400 000. Brown green beads represent PES; orange beads represent the PES segments in the PES-*b*-PCBMA copolymer; and cyan depicts MMA segments (composed of A). Solvent D beads, water beads, and zwitterionic segment B beads are omitted for clarity. Adapted from ref (220). Copyright 2019 American Chemical Society. (c) Molecular structures of the 12 zwitterionic moieties studied. Adapted from ref (226). Copyright 2014 American Chemical Society. (d) (Left panel) One Levodopa molecule in the POPC-cholesterol bilayer. All the cholesterol molecules in the visualized lipid phase are depicted in the color blue, and again the Levodopa is portrayed in black color with green contour. (Right panel) Mass density profiles of the aqueous phase, cholesterol-free lipid phase consisting of DPPC molecules and Levodopa in its zwitterionic form along the normal to the two leaflets of the bilayer. Adapted with permission from ref (113). Copyright 2021 Elsevier.

Understanding the role of surface modification on nanoparticle–biomembrane interactions is crucial in promoting the application of nanoparticles in biomedical fields.^217^ Quan *et al.*(160) investigated the interactions between polymer-coated gold nanoparticles (AuNPs) and lipid membranes using CG-MD simulations. The results showed that grafting AuNPs with zwitterionic polymers facilitated the nanoparticles’ approach to the membrane surface compared to those grafted with hydrophilic PEG. They also found that, for zwitterionic polymer-coated AuNPs, which could undergo pH-dependent charge conversion, the degree of polymer protonation impacted different interaction modes. At a low protonation degree, particle adsorption on the membrane surface occurred, while at a moderate degree of protonation particle translocation across the lipid membrane was observed. The curved lipid membrane entirely wrapped the particles at high protonation degrees, leading to endocytosis. They also studied the effect of polymer chain length on the cellular uptake of zwitterionic polymer-coated AuNPs. Their results demonstrated that when the protonation degree was not high, the long polymer chains would block the translocation of AuNPs across the lipid membrane. However, the long chain length could improve the transmembrane efficiency of AuNPs at high protonation degrees (see Figure 7a). These findings are expected to be of strong interest for the design and synthesis of pH-responsive nanomaterials based on polyzwitterions and prompts their further applications in the field of biomedicine and drug delivery. This type of coarse-graining research is a good example that provides regulations for designing other surface-functionalized nanoparticles, including different possible inorganic/organic particles and novel (poly)zwitterions as drug delivery systems.

Kovacevic *et al.*(218) performed MD simulations to study how the size, hydrophobicity, and drug concentration affect the structure of zwitterion functionalized AuNPs. They simulated two groups of nanosystems functionalized with a zwitterion and a ligand carrying a drug. They showed that in the case of a hydrophobic drug, the hydrophobicity controlled the conformational changes of the coating layer. In the case of a hydrophilic drug, the final structure of the coating conformations was controlled by the ligands. The results also showed that the percentage of the accessible hydrophilic drug was remarkably higher than in the hydrophobic systems. It implies that higher biological efficiency can be expected for hydrophilic systems. This research highlights the importance of taking into account physicochemical properties of drugs and ligands at the atomistic level (see Table 2 for the scales that MD covers) when developing gold-based nanosystems, especially in the case of hydrophobic drugs.

### 2.3 Membrane

For most drugs, regardless of the dosage form and the administration route, a crucial step in generating a biological effect is represented by the interaction of the drug with a receptor that can be located either on the cell membrane or inside the cell.^219^ Therefore, one part of the drug delivery process is passing drugs or drug delivery systems across cell membranes. Due to the similarity between lipid bilayer zwitterionic membranes and biological cell membranes, monitoring (1) interactions between drugs and zwitterionic membranes, (2) drug transport properties over zwitterionic membranes, and (3) drug orientation and accumulation in the membranes, can provide useful information for developing novel drug delivery systems. Here, one question is how molecular modeling of zwitterionic membranes can assist this development process. Modeling of different drug delivery systems (for instance, zwitterionic-based drug delivery) passing through zwitterionic membranes can represent a model of drug carriers penetrating the cell. The atomistic level information on this process can provide a better perspective on how to design intelligent drug delivery systems (*i.e.*, engineering the size, shape, surface functionality, stability, internal structure, drug encapsulation level) with higher cellular uptake efficacy (see Figure 4). In the research field of zwitterion membranes, Huo *et al.*(220) adopted DPD simulations to investigate the nonsolvent induced phase separation (NIPS) process during a pH-responsive poly(ether sulfone) membrane preparation with a zwitterionic copolymer poly(ether sulfone)-*block*-polycarboxybetaine methacrylate (PES-*b*-PCBMA) as the blending additive. Simulation results revealed that the hydrophilic PCBMA segments enriched on the membrane surface by surface segregation (see Figure 7b) and exhibited pH-responsive behavior, which was attributed to the deprotonation of carboxylic acid groups. With the polymer concentration increasing, both the membrane shrinkage and the system’s flexibility decreased, which in turn reduced the effect of surface segregation. Their research work contributes to a better understanding of the mechanism of NIPS and can provide a guide to designing a wide range of novel zwitterion-based polymer bi- or multicomponent blend membranes, considering features of each component (chemistry and architecture) individually and noncovalent interactions in blended systems with crosstalk.

Significant efforts have been directed to develop a fundamental understanding of (poly)zwitterion surface antifouling mechanisms at the molecular level.^221,222^ He *et al.*(14) performed molecular simulations to study the interactions of a model protein (LYZ) with a phosphorylcholine-terminated self-assembled monolayer (PC-SAM) surface in the presence of explicit water molecules and ions. The results were compared with those obtained for an oligo(ethylene glycol) terminated self-assembled monolayer (OEG-SAM) surface and bulk water. Using the radial distribution function and the residence time dynamics analysis, they showed the hydration layer of the zwitterionic PC-SAM to be stronger than that of OEG-SAM. The water molecules above the PC-SAM surface repelled the protein robustly as it approached the surface, which was in good agreement with previous experimental studies, confirming the nonfouling nature of PC-SAM surfaces.^223,224^ In spite of the antifouling feature of both PC-SAM and OEG-SAM surfaces, the zwitterionic PC-SAM surface was found to be entirely different from the OEG-SAM surface. First, comparing the hydration layer residence time between PC-SAM and OEG-SAM surfaces revealed a longer stay for water molecules in the hydration layer near an PC-SAM surface than an OEG-SAM surface. This indicates that the PC-SAM surface binds water molecules more tightly compared to the OEG-SAM surface. Second, the water molecules near the PC-SAM surface had a dipole distribution that was much closer to bulk water than on the OEG-SAM surface. Finally, the interfacial water molecules near the PC-SAM surface, which did not form hydrogen bonds with the PC chains, had reorientational dynamics similar to those of bulk water molecules but was much slower than those near the OEG-SAM surface. Despite these differences, hydration still played a key role in the antiprotein adsorption of PC-SAM surfaces.^14^ From a technical viewpoint, this research shows that the accuracy, time, and length scale of the MD method is appropriate to capture molecular-level information on protein-monolayer surfaces (see the time and length scale of MD modeling in Table 2). The information includes nonfouling mechanisms and details for (poly)zwitterions (or other antifouling agents), formation of a water hydration layer, orientation of water and fouling agent (*i.e.*, protein), and noncovalent interaction molecules. In principle, the selection of simulation techniques to study a system depends on the depth and scale of the required information.

In the anti(bio)fouling field Shao *et al.*,^225^ studied the interactions between carboxybetaine (CB) solution and chymotrypsin inhibitor 2 (CI2) and the effect of the zwitterion on the structure of the protein, using MD simulations. They also compared the structural properties of CI2 in CB and oligo(ethylene glycol) (OEG) solutions. The simulation results indicated that zwitterionic CB and nonionic OEG moieties did not accumulate around the surface of the CI2, confirming the anti(bio)fouling behavior of both of them. They also indicated that although the protein could retain its folded structure in both CB and OEG solutions, superhydrophobic CB had a minimal effect on the protein. This observation is attributed to the zwitterionic nature of both CB and CI2, whereas amphiphilic OEG changed the properties of the protein via hydrophobic interactions.^225^ In another research, Shao and Jiang^226^ studied the roles of charged groups in zwitterions to design new anti(bio)fouling (protein-resistant) zwitterionic moieties beyond carboxybetaine and sulfobetaine. They studied the hydration and protein interactions of 12 zwitterions (see Figure 7c) derived from three anionic groups (carboxylic, sulfonate, and sulfate) and four cationic groups (quaternary ammonium, tertiary ammonium, secondary ammonium, and primary ammonium) via molecular simulations. They studied hydration level by evaluating the hydration-free energy of zwitterions and the hydration structure and dynamics of the charged groups. They showed that all zwitterions had strong hydration, but their structural and dynamic properties depended on the type of cationic and anionic groups. Let us discuss the importance of this research type in designing intelligent drug delivery platforms. One of the main hurdles to drug delivery systems is that bioadhesion (*i.e*., protein adhesion) controls the fate of drug transporter *in vivo* and makes the interface between proteins and biological surfaces, influencing their physiological response like cellular uptake and targeting efficiency. Therefore, engineering the drug delivery systems using anti(bio)fouling agents is an extremely important issue for designing useful diagnostic and therapeutic systems. This type of research can be an efficient approach to study different anti(bio)fouling agents (*i.e.*, various types of (poly)zwitterions) for application in in drug vehicles to screen their interaction with the environment and to monitor their antifouling performance efficacy.

In recent work, Megariotis *et al.*(113) presented MD and umbrella sampling simulations of the levodopa zwitterion formulations (standard medication for Parkinson’s disease^227^) at various concentrations in between two hydrated zwitterion bilayers formed with cholesterol-free 1,2-dipalmitoyl-*sn*-glycero-3-phosphocholine (DPPC) and cholesterol-containing 1-palmitoyl-2-oleoyl-*sn*-glycero-3-phosphocholine (POPC), respectively (Figure 7d). They chose these systems because, in an effective treatment process, Levodopa has to cross the blood-brain barrier (BBB), with the help of the membrane protein LAT1 (L-type amino acid transporter 1), to be converted to dopamine.^228^ They aimed to investigate the extent to which Levodopa can be transported passively through the BBB. The main purpose of their research was to study Levodopa’s behavior in different hydrated lipid membranes, mainly from the thermodynamic point of view, and elucidate features of Levodopa’s permeation mechanism through the studied bilayer membranes. To screen Levodopa’s permeability through the membranes, local concentration, and orientation in the membranes, they calculated self-diffusion coefficients, mass density profiles, and order parameters. Their calculations of the potentials of mean force by umbrella sampling simulations revealed that Levodopa zwitterions, which formed a network of hydrogen bonds with water and phospholipid molecules, are found to be preferentially located at the water/lipid interface (see the density profile curve in Figure 7d).^113^ Through this research, one can find out the practicality of a combination of biased and unbiased simulation techniques in studying drug delivery. For example, the umbrella sampling simulations constitute free energy profiles that explain how the drug behaves in the heterogeneous biological systems considered herein. On the other hand, unbiased simulations allow us to compute properties such as mass density profiles, self-diffusion coefficients, and specific order parameters. The applied strategy in this research, as a powerful tool for the *in silico* study of drugs in different lipid environments, provides information and deep insight at the nanoscopic level that is not easily extracted from *in vivo*, *ex vivo*, or *in vitro* experiments.

## 3 Conclusion, Perspectives, and Future Outlook

Herein, we have reviewed the current state-of-the-art and research works on (poly)zwitterion modeling for potential drug delivery applications, identifying the techniques required for predictive modeling-led design. (Poly)zwitterions are fascinating soft materials with a wide range of structures and chemistries. Their unique properties, such as antifouling, make them very interesting for a plethora of applications in (bio)medicine. This Perspective aims to amplify the importance of modeling-led research for the characterization of (poly)zwitterionic materials, which will enable the predictive design and surface engineering required for the self-assembly of more suitable drug delivery platforms.

Let us summarize the importance of (poly)zwitterionic self-assembly and surface engineering-led zwitterionic materials design for drug delivery, which reveals the necessity of understanding this type of systems at the atomic level. Drug encapsulation by polyzwitterions can occur through drug adsorption in the core of the formed micelle or the vesicle’s aqueous core or through drug conjugation on the aforementioned self-assembled particle surface. However, the (poly)zwitterion self-assembled structure’s size, shape, and internal structure are attributed to the polymer chemical composition, chain length, and architecture. The parameters that control the structure of (poly)zwitterions can independently or cooperatively govern the self-assembled formation and structure and thus the drug delivery pathways. Understanding the impact of the structural parameters on self-assembled particle-mediated drug delivery can teach us how to design intelligent multifunctional (poly)zwitterion-based drug vehicles. We also discussed the role of zwitterionic lipid bilayers, since these mimic biological cell membranes that control the flow of substances in and out of the cell. Modeling this type of membrane is essential since the information obtained from different molecular modeling techniques about transport properties and mechanisms of drugs across the membranes can shed light on this complicated biological process and improve drug pharmacokinetics by screening drug-membrane interactions and drug orientation. Finally, modification of drug delivery nanoparticles’ physicochemical properties via surface functionalization by zwitterions was investigated. Modifying surfaces with zwitterionic materials leads to an adjustment in charge densities, optimizing the solubility and increasing the stability in biological fluids, thus increasing targeted uptake.

We envision that this introductory text will catalyze the understanding and design criteria for (poly)zwitterions and allow for their translation into more real-world applications. In the field of polymer science, the development of advanced materials is currently one of the main challenges. In our opinion, the future of intelligent (poly)zwitterions-based drug delivery system lies in

### 3.1 Composite Materials

Novel hybrid platforms synthesized via self-assembly of a composite of (poly)zwitterions and inorganic smart nanoparticles, such as inorganic dendrimers and organic–inorganic hybrids, can be optimizedto carry drugs owing to their unique properties such as controllable interactions with biological material, useful electronic, magnetic, and optical properties. To design novel composites for drug delivery, predictive modeling-based studies, from atomistic level to coarse-graining, are recommended prior to large-scale experimental studies. This will shed light on the roadmap for the design of composite materials leading toward more targeted experiments.

### 3.2 Synthesizing Polymer with New Chemical Structure

To build intelligent (poly)zwitterion-based vesicles, rationally engineering novel (poly)zwitterion chemical structures can be necessary. To this end (poly)zwitterions can be functionalized or grafted with conjugated molecules, biomolecules, noncharged polymers with different architectures, or inorganic nanostructures. The new polymer compositions may self-assemble into new types of supramolecular structures with potentially valuable functions in biomedicine. Morphological phase diagrams of the supramolecular structures can be built using molecular modeling, particularly coarse-graining techniques. Having modeling-led morphological phase diagrams is recommended to direct experimental studies, including synthesis and functionalization, considering the required structure for drug vehicles.

### 3.3 Multicomponent Systems

The capacity of intelligent (poly)zwitterions-based drug vehicles can be grown up from binary systems to multicomponent systems, taking advantage of the unique properties of each component. Based on the structure and properties that are required from a drug delivery platform, all the components can be designed by, for instance, altering the repeating units or copolymerization. This process can be accelerated by employing appropriate computational screening studies. The road from design to novel applications of multicomponent (poly)zwitterion materials is long, and can be shortened if aided by multiscale modeling. The reasons can be attributed to the philosophy about macromolecule models and simulations (from atomistic to mesoscale) which situates itself somewhere between the domain of chemistry (which is led by work with atomistic detail) and physics (design general models to describe different phenomena).

### 3.4 Diverse Architectures

Using smart multifunctional copolymer/(poly)zwitterions combinations or other smart polymers (*e.g.*, polyelectrolytes and thermosensitive polymers)/(poly)zwitterions may produce novel classes of architecture for intelligent drug vehicles. By changing the parameters of the components (relative concentration, chemistry, composition, size, molecular symmetry, and mechanical/thermal properties), they can be successfully engineered. Different multifunctional zwitterionic smart copolymers with various architectures, including star-like, dendritic, hyperbranched, comb-like, cyclic, and H-shaped, whose potential in drug delivery is unknown, can be designed. Molecular dynamics simulationscan show us the atomistic aspects of the designed multifunctional molecules, while their performance in the drug delivery process, drug encapsulation or conjugation, stability, and release can be understood via mesoscale modeling. Summarizing, all the effective parameters controlling the drug-carrying can be studied via multiscale molecular simulations to deliver a comprehensive guide and shortest route to experimental validation to shortest routes experiments.

### 3.5 Other Therapeutics

The application horizon of existing state-of-the-art polyzwitterion/drug formulations can be expanded to other therapeutics (antibodies, proteins, peptides, and vitamins, *etc.*). Furthermore, the zwitterionic capsules can also be specifically designed and optimized for different administration routes. To change the administration route of the aforementioned therapeutics to more convenient methods, modeling-led studies are recommended for the following reasons: to design suitable polyzwitterion molecules, to monitor if the molecule can respond to stimuli in the desired new administration route properly, to track the supramolecular formation of polyzwitterion/therapeutics at the molecular level, to learn about the roles of different interactions in the system to boost the more favorable interactions contributions, and to decrease the number of required experiments by using modeling-guided protocols.

### 3.6 Modeling Advances

The theoretical development and the range of applications of molecular modeling are expanding rapidly. However, there is still a long way until force fields become so reliable as to implement modeling-guided studies with experimental accuracy. In this regard, machine learning approaches can be helpful since their recent tremendous success has proven that they can also lead to great advances in the developing force fields. Some of these force fields may assist us to push the modeling of polymeric drug delivery forward to simulate the events taking place at longer time scales than we can currently simulate using coarse-grained molecular techniques.

### 3.7 Other Applications

Taking full advantage of intelligent (poly)zwitterions and their unique characteristics, they can be employed in other applications, such as separation materials, sensors, catalysts, *etc.* The responsiveness shown by polyzwitterions is specific, sensitive, and instantaneous. The future challenge lies in engineering this class of materials for hybrid material applications, controlled drug biomedical devices, *etc.* In this regard, predictive multiscale molecular modeling studies can smooth the complicated road of designing novel (poly)zwitterion-based materials and exploiting them in different applications. On the other hand, modeling approaches provide explanations of experimentally observed molecular structure, dynamics, thermodynamics, and zwitterionic material properties at microscopic and macroscopic scale.

## References

[ref1] Hess M.; Jones R. G.; Kahovec J.; Kitayama T.; Kratochvíl P.; Kubisa P.; Mormann W.; Stepto R. F. T.; Tabak D.; Vohlídal J.; Wilks E. S. Terminology of polymers containing ionizable or ionic groups and of polymers containing ions (IUPAC Recommendations 2006). Pure Appl. Chem. 2006, 78 (11), 2067–2074. 10.1351/pac200678112067.

[ref2] Blackman L. D.; Gunatillake P. A.; Cass P.; Locock K. E. S. An Introduction to Zwitterionic Polymer Behavior and Applications in Solution and at Surfaces. Chem. Soc. Rev. 2019, 48 (3), 757–770. 10.1039/C8CS00508G. 30548039

[ref3] Harijan M.; Singh M. Zwitterionic Polymers in Drug Delivery: A Review. J. Mol. Recognit 2022, 35 (1), e2944 10.1002/jmr.2944. 34738272

[ref4] Mahalik J. P.; Muthukumar M. Simulation of Self-Assembly of Polyzwitterions into Vesicles. J. Chem. Phys. 2016, 145 (7), 074907 10.1063/1.4960774. 27544126

[ref5] Zhou L.-Y.; Zhu Y.-H.; Wang X.-Y.; Shen C.; Wei X.-W.; Xu T.; He Z.-Y. Novel Zwitterionic Vectors: Multi-Functional Delivery Systems for Therapeutic Genes and Drugs. Computational and Structural Biotechnology Journal 2020, 18, 1980–1999. 10.1016/j.csbj.2020.07.015. 32802271 PMC7403891

[ref6] Laschewsky A. Structures and Synthesis of Zwitterionic Polymers. Polymers 2014, 6 (5), 1544–1601. 10.3390/polym6051544.

[ref7] Mark H. F., Ed. Encyclopedia of Polymer Science and Technology, 15 Vol. Set, 4th ed.; Wiley, 2014. https://www.wiley.com/en-us/Encyclopedia+of+Polymer+Science+and+Technology%2C+15+Vol.+Set%2C+4th+Edition-p-9781118633892 (accessed 2021-12-02).

[ref8] Lowe A. B.; McCormick C. L. Synthesis and Solution Properties of Zwitterionic Polymers. Chem. Rev. 2002, 102 (11), 4177–4190. 10.1021/cr020371t. 12428987

[ref9] Kudaibergenov S.; Jaeger W.; Laschewsky A. Polymeric Betaines: Synthesis, Characterization, and Application. In Supramolecular Polymers Polymeric Betains Oligomers; Advances in Polymer Science; Springer: Berlin, Heidelberg, 2006; pp 157–224. 10.1007/12_078.

[ref10] Xu S.; Nilles J. M.; Bowen K. H. Zwitterion Formation in Hydrated Amino Acid, Dipole Bound Anions: How Many Water Molecules Are Required?. J. Chem. Phys. 2003, 119 (20), 10696–10701. 10.1063/1.1620501.

[ref11] Laughlin R. G. Fundamentals of the Zwitterionic Hydrophilic Group. Langmuir 1991, 7 (5), 842–847. 10.1021/la00053a006.

[ref12] Shao Q.; Jiang S. Molecular Understanding and Design of Zwitterionic Materials. Adv. Mater. 2015, 27 (1), 15–26. 10.1002/adma.201404059. 25367090

[ref13] Zou H.; Wang Z.; Feng M. Nanocarriers with Tunable Surface Properties to Unblock Bottlenecks in Systemic Drug and Gene Delivery. J. Controlled Release 2015, 214, 121–133. 10.1016/j.jconrel.2015.07.014. 26208425

[ref14] He Y.; Hower J.; Chen S.; Bernards M. T.; Chang Y.; Jiang S. Molecular Simulation Studies of Protein Interactions with Zwitterionic Phosphorylcholine Self-Assembled Monolayers in the Presence of Water. Langmuir 2008, 24 (18), 10358–10364. 10.1021/la8013046. 18690732

[ref15] Schlenoff J. B. Zwitteration: Coating Surfaces with Zwitterionic Functionality to Reduce Nonspecific Adsorption. Langmuir 2014, 30 (32), 9625–9636. 10.1021/la500057j. 24754399 PMC4140545

[ref16] Ladenheim H.; Morawetz H. A New Type of Polyampholyte: Poly(4-Vinyl Pyridine Betaine). J. Polym. Sci. 1957, 26 (113), 251–254. 10.1002/pol.1957.1202611319.

[ref17] Hart R.; Timmerman D. New Polyampholytes: The Polysulfobetaines. J. Polym. Sci. 1958, 28 (118), 638–640. 10.1002/pol.1958.1202811820.

[ref18] Xiao S.; Ren B.; Huang L.; Shen M.; Zhang Y.; Zhong M.; Yang J.; Zheng J. Salt-Responsive Zwitterionic Polymer Brushes with Anti-Polyelectrolyte Property. Current Opinion in Chemical Engineering 2018, 19, 86–93. 10.1016/j.coche.2017.12.008.

[ref19] Xiao S.; Zhang Y.; Shen M.; Chen F.; Fan P.; Zhong M.; Ren B.; Yang J.; Zheng J. Structural Dependence of Salt-Responsive Polyzwitterionic Brushes with an Anti-Polyelectrolyte Effect. Langmuir 2018, 34 (1), 97–105. 10.1021/acs.langmuir.7b03667. 29232140

[ref20] Sgouros A. P.; Knippenberg S.; Guillaume M.; Theodorou D. N. Multiscale Simulations of Polyzwitterions in Aqueous Bulk Solutions and Brush Array Configurations. Soft Matter 2021, 17 (48), 10873–10890. 10.1039/D1SM01255J. 34807216

[ref21] Tang Y.; Lu J. R.; Lewis A. L.; Vick T. A.; Stratford P. W. Swelling of Zwitterionic Polymer Films Characterized by Spectroscopic Ellipsometry. Macromolecules 2001, 34 (25), 8768–8776. 10.1021/ma010476i.

[ref22] Wei R.; Song W.; Yang F.; Zhou J.; Zhang M.; Zhang X.; Zhao W.; Zhao C. Bidirectionally PH-Responsive Zwitterionic Polymer Hydrogels with Switchable Selective Adsorption Capacities for Anionic and Cationic Dyes. Ind. Eng. Chem. Res. 2018, 57 (24), 8209–8219. 10.1021/acs.iecr.8b01027.

[ref23] Wu J.; He C.; He H.; Cheng C.; Zhu J.; Xiao Z.; Zhang H.; Li X.; Zheng J.; Xiao J. Importance of Zwitterionic Incorporation into Polymethacrylate-Based Hydrogels for Simultaneously Improving Optical Transparency, Oxygen Permeability, and Antifouling Properties. J. Mater. Chem. B 2017, 5 (24), 4595–4606. 10.1039/C7TB00757D. 32264302

[ref24] Cao B.; Tang Q.; Li L.; Humble J.; Wu H.; Liu L.; Cheng G. Switchable Antimicrobial and Antifouling Hydrogels with Enhanced Mechanical Properties. Adv. Healthcare Mater. 2013, 2 (8), 1096–1102. 10.1002/adhm.201200359. 23386310

[ref25] Liu J.; Yang K.; Shao W.; Li S.; Wu Q.; Zhang S.; Qu Y.; Zhang L.; Zhang Y. Synthesis of Zwitterionic Polymer Particles via Combined Distillation Precipitation Polymerization and Click Chemistry for Highly Efficient Enrichment of Glycopeptide. ACS Appl. Mater. Interfaces 2016, 8 (34), 22018–22024. 10.1021/acsami.6b06343. 27498760

[ref26] Durrani A. A.; Hayward J. A.; Chapman D. Biomembrnanes as Models for Polymer Surfaces: II. The Syntheses of Reactive Species for Covalent Coupling of Phosphorylcholine to Polymer Surfaces. Biomaterials 1986, 7 (2), 121–125. 10.1016/0142-9612(86)90068-2. 3708063

[ref27] Ventura C.; Guerin A. J.; El-Zubir O.; Ruiz-Sanchez A. J.; Dixon L. I.; Reynolds K. J.; Dale M. L.; Ferguson J.; Houlton A.; Horrocks B. R.; Clare A. S.; Fulton D. A. Marine Antifouling Performance of Polymer Coatings Incorporating Zwitterions. Biofouling 2017, 33 (10), 892–903. 10.1080/08927014.2017.1383983. 29083230

[ref28] Xiao S.; Zhang M.; He X.; Huang L.; Zhang Y.; Ren B.; Zhong M.; Chang Y.; Yang J.; Zheng J. Dual Salt- and Thermoresponsive Programmable Bilayer Hydrogel Actuators with Pseudo-Interpenetrating Double-Network Structures. ACS Appl. Mater. Interfaces 2018, 10 (25), 21642–21653. 10.1021/acsami.8b06169. 29878750

[ref29] Maji T.; Banerjee S.; Biswas Y.; Mandal T. K. Dual-Stimuli-Responsive l-Serine-Based Zwitterionic UCST-Type Polymer with Tunable Thermosensitivity. Macromolecules 2015, 48 (14), 4957–4966. 10.1021/acs.macromol.5b01099.

[ref30] Chen M.; Briscoe W. H.; Armes S. P.; Cohen H.; Klein J. Polyzwitterionic Brushes: Extreme Lubrication by Design. Eur. Polym. J. 2011, 47 (4), 511–523. 10.1016/j.eurpolymj.2010.10.007.

[ref31] Kobayashi M.; Terayama Y.; Hosaka N.; Kaido M.; Suzuki A.; Yamada N.; Torikai N.; Ishihara K.; Takahara A. Friction Behavior of High-Density Poly(2-Methacryloyloxyethyl Phosphorylcholine) Brush in Aqueous Media. Soft Matter 2007, 3 (6), 740–746. 10.1039/b615780g. 32900137

[ref32] Bai T.; Liu S.; Sun F.; Sinclair A.; Zhang L.; Shao Q.; Jiang S. Zwitterionic Fusion in Hydrogels and Spontaneous and Time-Independent Self-Healing under Physiological Conditions. Biomaterials 2014, 35 (13), 3926–3933. 10.1016/j.biomaterials.2014.01.077. 24559638

[ref33] Chou Y.-N.; Sun F.; Hung H.-C.; Jain P.; Sinclair A.; Zhang P.; Bai T.; Chang Y.; Wen T.-C.; Yu Q.; Jiang S. Ultra-Low Fouling and High Antibody Loading Zwitterionic Hydrogel Coatings for Sensing and Detection in Complex Media. Acta Biomaterialia 2016, 40, 31–37. 10.1016/j.actbio.2016.04.023. 27090589

[ref34] Yang W.; Bai T.; Carr L. R.; Keefe A. J.; Xu J.; Xue H.; Irvin C. A.; Chen S.; Wang J.; Jiang S. The Effect of Lightly Crosslinked Poly(Carboxybetaine) Hydrogel Coating on the Performance of Sensors in Whole Blood. Biomaterials 2012, 33 (32), 7945–7951. 10.1016/j.biomaterials.2012.07.035. 22863377

[ref35] Brown R. H.; Duncan A. J.; Choi J.-H.; Park J. K.; Wu T.; Leo D. J.; Winey K. I.; Moore R. B.; Long T. E. Effect of Ionic Liquid on Mechanical Properties and Morphology of Zwitterionic Copolymer Membranes. Macromolecules 2010, 43 (2), 790–796. 10.1021/ma902028u.

[ref36] Wu T.; Beyer F. L.; Brown R. H.; Moore R. B.; Long T. E. Influence of Zwitterions on Thermomechanical Properties and Morphology of Acrylic Copolymers: Implications for Electroactive Applications. Macromolecules 2011, 44 (20), 8056–8063. 10.1021/ma201211j.

[ref37] Tan X.; Duan L.; Han W.; Li Y.; Guo M. A Zwitterionic Copolymer as Rheology Modifier and Fluid Loss Agents for Water-Based Drilling Fluids. Polymers 2021, 13 (18), 3120 10.3390/polym13183120. 34578021 PMC8471074

[ref38] Wang J.; Feng Y.; Agrawal N. R.; Raghavan S. R. Wormlike Micelles versus Water-Soluble Polymers as Rheology-Modifiers: Similarities and Differences. Phys. Chem. Chem. Phys. 2017, 19 (36), 24458–24466. 10.1039/C7CP04962E. 28880323

[ref39] Jin Q.; Chen Y.; Wang Y.; Ji J. Zwitterionic Drug Nanocarriers: A Biomimetic Strategy for Drug Delivery. Colloids Surf., B 2014, 124, 80–86. 10.1016/j.colsurfb.2014.07.013. 25092584

[ref40] Li B.; Yuan Z.; Zhang P.; Sinclair A.; Jain P.; Wu K.; Tsao C.; Xie J.; Hung H.-C.; Lin X.; Bai T.; Jiang S. Zwitterionic Nanocages Overcome the Efficacy Loss of Biologic Drugs. Adv. Mater. 2018, 30 (14), e1705728 10.1002/adma.201705728. 29457278

[ref41] van Meer G.; Voelker D. R.; Feigenson G. W. Membrane Lipids: Where They Are and How They Behave. Nat. Rev. Mol. Cell Biol. 2008, 9 (2), 112–124. 10.1038/nrm2330. 18216768 PMC2642958

[ref42] Binazadeh M.; Kabiri M.; Unsworth L. D. Poly(ethylene glycol) and Poly(carboxy betaine) Based Nonfouling Architectures: Review and Current Efforts. In Proteins at Interfaces III State of the Art; ACS Symposium Series; American Chemical Society, 2012; Vol. 1120, pp 621–643. 10.1021/bk-2012-1120.ch028.

[ref43] Lopez-Donaire M. L.; Santerre J. P. Surface Modifying Oligomers Used to Functionalize Polymeric Surfaces: Consideration of Blood Contact Applications. J. Appl. Polym. Sci. 2014, 131 (14), 40328 10.1002/app.40328.

[ref44] Rampado R.; Crotti S.; Caliceti P.; Pucciarelli S.; Agostini M. Recent Advances in Understanding the Protein Corona of Nanoparticles and in the Formulation of “Stealthy” Nanomaterials. Front Bioeng Biotechnol 2020, 8, 166 10.3389/fbioe.2020.00166. 32309278 PMC7145938

[ref45] Liu Y.; Zhang D.; Ren B.; Gong X.; Xu L.; Feng Z.-Q.; Chang Y.; He Y.; Zheng J. Molecular Simulations and Understanding of Antifouling Zwitterionic Polymer Brushes. J. Mater. Chem. B 2020, 8 (17), 3814–3828. 10.1039/D0TB00520G. 32227061

[ref46] Liu J.; Zhou J. Hydrolysis-Controlled Protein Adsorption and Antifouling Behaviors of Mixed Charged Self-Assembled Monolayer: A Molecular Simulation Study. Acta Biomater 2016, 40, 23–30. 10.1016/j.actbio.2016.04.044. 27134014

[ref47] He M.; Gao K.; Zhou L.; Jiao Z.; Wu M.; Cao J.; You X.; Cai Z.; Su Y.; Jiang Z. Zwitterionic Materials for Antifouling Membrane Surface Construction. Acta Biomaterialia 2016, 40, 142–152. 10.1016/j.actbio.2016.03.038. 27025359

[ref48] Fan L.; Wang X.; Cao Q.; Yang Y.; Wu D. POSS-Based Supramolecular Amphiphilic Zwitterionic Complexes for Drug Delivery. Biomater. Sci. 2019, 7 (5), 1984–1994. 10.1039/C9BM00125E. 30834395

[ref49] Wu A.; Gao Y.; Zheng L. Zwitterionic Amphiphiles: Their Aggregation Behavior and Applications. Green Chem. 2019, 21 (16), 4290–4312. 10.1039/C9GC01808E.

[ref50] Han X.; Lu Y.; Xie J.; Zhang E.; Zhu H.; Du H.; Wang K.; Song B.; Yang C.; Shi Y.; Cao Z. Zwitterionic Micelles Efficiently Deliver Oral Insulin without Opening Tight Junctions. Nat. Nanotechnol. 2020, 15 (7), 605–614. 10.1038/s41565-020-0693-6. 32483319 PMC7534179

[ref51] George D. K.; Charkhesht A.; Hull O. A.; Mishra A.; Capelluto D. G. S.; Mitchell-Koch K. R.; Vinh N. Q. New Insights into the Dynamics of Zwitterionic Micelles and Their Hydration Waters by Gigahertz-to-Terahertz Dielectric Spectroscopy. J. Phys. Chem. B 2016, 120 (41), 10757–10767. 10.1021/acs.jpcb.6b06423. 27661395

[ref52] Zumbuehl A. Artificial Phospholipids and Their Vesicles. Langmuir 2019, 35 (32), 10223–10232. 10.1021/acs.langmuir.8b02601. 30278137

[ref53] McCoy T. M.; Marlow J. B.; Armstrong A. J.; Clulow A. J.; Garvey C. J.; Manohar M.; Darwish T. A.; Boyd B. J.; Routh A. F.; Tabor R. F. Spontaneous Self-Assembly of Thermoresponsive Vesicles Using a Zwitterionic and an Anionic Surfactant. Biomacromolecules 2020, 21 (11), 4569–4576. 10.1021/acs.biomac.0c00672. 32597638

[ref54] Lombardo D.; Kiselev M.; magazù S.; Calandra P. Amphiphiles Self-Assembly: Basic Concepts and Future Perspectives of Supramolecular Approaches. Advances in Condensed Matter Physics 2015, 2015, 1–22. 10.1155/2015/151683.

[ref55] He Q.; Yan R.; Hou W.; Wang H.; Tian Y. A PH-Responsive Zwitterionic Polyurethane Prodrug as Drug Delivery System for Enhanced Cancer Therapy. Molecules 2021, 26 (17), 5274 10.3390/molecules26175274. 34500707 PMC8434572

[ref56] Gao S.; Holkar A.; Srivastava S. Protein–Polyelectrolyte Complexes and Micellar Assemblies. Polymers 2019, 11 (7), 1097 10.3390/polym11071097. PMC6680422 31261765

[ref57] Khan N.; Brettmann B. Intermolecular Interactions in Polyelectrolyte and Surfactant Complexes in Solution. Polymers (Basel) 2019, 11 (1), 51 10.3390/polym11010051. PMC6401804 30960035

[ref58] Kawakami K.; Ebara M.; Izawa H.; Sanchez-Ballester N. M.; Hill J. P.; Ariga K. Supramolecular Approaches for Drug Development. Curr. Med. Chem. 2012, 19 (15), 2388–2398. 10.2174/092986712800269254. 22455591

[ref59] Webber M. J.; Langer R. Drug Delivery by Supramolecular Design. Chem. Soc. Rev. 2017, 46 (21), 6600–6620. 10.1039/C7CS00391A. 28828455

[ref60] Soni S. S.; Alsasa A.; Rodell C. B. Applications of Macrocyclic Host Molecules in Immune Modulation and Therapeutic Delivery. Front. Chem. 2021, 9, 658548 10.3389/fchem.2021.658548. 33889565 PMC8055865

[ref61] Bojarska J.; Remko M.; Breza M.; Madura I. D.; Kaczmarek K.; Zabrocki J.; Wolf W. M. A Supramolecular Approach to Structure-Based Design with A Focus on Synthons Hierarchy in Ornithine-Derived Ligands: Review, Synthesis, Experimental and in Silico Studies. Molecules 2020, 25 (5), 1135 10.3390/molecules25051135. PMC7179192 32138329

[ref62] Contini C.; Pearson R.; Wang L.; Messager L.; Gaitzsch J.; Rizzello L.; Ruiz-Perez L.; Battaglia G. Bottom-Up Evolution of Vesicles from Disks to High-Genus Polymersomes. iScience 2018, 7, 132–144. 10.1016/j.isci.2018.08.018. 30267675 PMC6153420

[ref63] Su C.-J.; Lee M.-T.; Liao K.-F.; Shih O.; Jeng U.-S. Interplay of Entropy and Enthalpy in Peptide Binding to Zwitterionic Phospholipid Membranes as Revealed from Membrane Thinning. Phys. Chem. Chem. Phys. 2018, 20 (42), 26830–26836. 10.1039/C8CP02861C. 30137074

[ref64] Marsh D. Thermodynamics of Phospholipid Self-Assembly. Biophys. J. 2012, 102 (5), 1079–1087. 10.1016/j.bpj.2012.01.049. 22404930 PMC3296042

[ref65] Weng J.; Yang M.; Wang W.; Xu X.; Tian Z. Revealing Thermodynamics and Kinetics of Lipid Self-Assembly by Markov State Model Analysis. J. Am. Chem. Soc. 2020, 142 (51), 21344–21352. 10.1021/jacs.0c09343. 33314927

[ref66] Sikorska E.; Wyrzykowski D.; Szutkowski K.; Greber K.; Lubecka E. A.; Zhukov I. Thermodynamics, Size, and Dynamics of Zwitterionic Dodecylphosphocholine and Anionic Sodium Dodecyl Sulfate Mixed Micelles. J. Therm Anal Calorim 2016, 123 (1), 511–523. 10.1007/s10973-015-4918-0.

[ref67] Zhang J. D.; Sach-Peltason L.; Kramer C.; Wang K.; Ebeling M. Multiscale Modelling of Drug Mechanism and Safety. Drug Discovery Today 2020, 25 (3), 519–534. 10.1016/j.drudis.2019.12.009. 31899257

[ref68] Amaro R. E.; Mulholland A. J. Multiscale Methods in Drug Design Bridge Chemical and Biological Complexity in the Search for Cures. Nat. Rev. Chem. 2018, 2 (4), 1–12. 10.1038/s41570-018-0148. PMC6445369 30949587

[ref69] Olson A. J. Perspectives on Structural Molecular Biology Visualization: From Past to Present. J. Mol. Biol. 2018, 430 (21), 3997–4012. 10.1016/j.jmb.2018.07.009. 30009769 PMC6186497

[ref70] Javan Nikkhah S.; Thompson D. Molecular Modelling Guided Modulation of Molecular Shape and Charge for Design of Smart Self-Assembled Polymeric Drug Transporters. Pharmaceutics 2021, 13 (2), 141 10.3390/pharmaceutics13020141. 33499130 PMC7912381

[ref71] Belviso F.; Claerbout V. E. P.; Comas-Vives A.; Dalal N. S.; Fan F.-R.; Filippetti A.; Fiorentini V.; Foppa L.; Franchini C.; Geisler B.; Ghiringhelli L. M.; Groß A.; Hu S.; Íñiguez J.; Kauwe S. K.; Musfeldt J. L.; Nicolini P.; Pentcheva R.; Polcar T.; Ren W.; Ricci F.; Ricci F.; Sen H. S.; Skelton J. M.; Sparks T. D.; Stroppa A.; Urru A.; Vandichel M.; Vavassori P.; Wu H.; Yang K.; Zhao H. J.; Puggioni D.; Cortese R.; Cammarata A. Viewpoint: Atomic-Scale Design Protocols toward Energy, Electronic, Catalysis, and Sensing Applications. Inorg. Chem. 2019, 58 (22), 14939–14980. 10.1021/acs.inorgchem.9b01785. 31668070

[ref72] Walden D. M.; Bundey Y.; Jagarapu A.; Antontsev V.; Chakravarty K.; Varshney J. Molecular Simulation and Statistical Learning Methods toward Predicting Drug–Polymer Amorphous Solid Dispersion Miscibility, Stability, and Formulation Design. Molecules 2021, 26 (1), 182 10.3390/molecules26010182. PMC7794704 33401494

[ref73] Aminpour M.; Montemagno C.; Tuszynski J. A. An Overview of Molecular Modeling for Drug Discovery with Specific Illustrative Examples of Applications. Molecules 2019, 24 (9), 1693 10.3390/molecules24091693. PMC6539951 31052253

[ref74] Fermeglia M.; Pricl S. Multiscale Molecular Modeling in Nanostructured Material Design and Process System Engineering. Comput. Chem. Eng. 2009, 33 (10), 1701–1710. 10.1016/j.compchemeng.2009.04.006.

[ref75] Posocco P.; Fermeglia M.; Pricl S. Morphology Prediction of Block Copolymers for Drug Delivery by Mesoscale Simulations. J. Mater. Chem. 2010, 20 (36), 7742–7753. 10.1039/c0jm01301c.

[ref76] Karayiannis N. Ch.; Mavrantzas V. G.; Theodorou D. N. A Novel Monte Carlo Scheme for the Rapid Equilibration of Atomistic Model Polymer Systems of Precisely Defined Molecular Architecture. Phys. Rev. Lett. 2002, 88 (10), 105503 10.1103/PhysRevLett.88.105503. 11909369

[ref77] Groenhof G. Introduction to QM/MM Simulations. In Biomolecular Simulations: Methods and Protocols; Monticelli L., Salonen E., Eds.; Methods in Molecular Biology; Humana Press: Totowa, NJ, 2013; pp 43–66. 10.1007/978-1-62703-017-5_3.

[ref78] Maximiano P.; Durães L.; Simões P. Overview of Multiscale Molecular Modeling and Simulation of Silica Aerogels. Ind. Eng. Chem. Res. 2019, 58 (41), 18905–18929. 10.1021/acs.iecr.9b03781.

[ref79] Mansfield K. F.; Theodorou D. N. Molecular dynamics simulation of a glassy polymer surface. Macromolecules 1991, 24, 6283–6294. 10.1021/ma00023a034.

[ref80] Hollingsworth S. A.; Dror R. O. Molecular Dynamics Simulation for All. Neuron 2018, 99 (6), 1129–1143. 10.1016/j.neuron.2018.08.011. 30236283 PMC6209097

[ref81] Karatrantos A.; Clarke N.; Kröger M. Modeling of Polymer Structure and Conformations in Polymer Nanocomposites from Atomistic to Mesoscale: A Review. Polym. Rev. 2016, 56 (3), 385–428. 10.1080/15583724.2015.1090450.

[ref82] Spyriouni T.; Tzoumanekas C.; Theodorou D.; Müller-Plathe F.; Milano G. Coarse-Grained and Reverse-Mapped United-Atom Simulations of Long-Chain Atactic Polystyrene Melts: Structure, Thermodynamic Properties, Chain Conformation, and Entanglements. Macromolecules 2007, 40 (10), 3876–3885. 10.1021/ma0700983.

[ref83] Shi X.; Tian F. Multiscale Modeling and Simulation of Nano-Carriers Delivery through Biological Barriers—A Review. Advanced Theory and Simulations 2019, 2 (1), 1800105 10.1002/adts.201800105.

[ref84] Joshi S. Y.; Deshmukh S. A. A Review of Advancements in Coarse-Grained Molecular Dynamics Simulations. Mol. Simul. 2021, 47 (10–11), 786–803. 10.1080/08927022.2020.1828583.

[ref85] Spijker P.; van Hoof B.; Debertrand M.; Markvoort A. J.; Vaidehi N.; Hilbers P. A. J. Coarse Grained Molecular Dynamics Simulations of Transmembrane Protein-Lipid Systems. Int. J. Mol. Sci. 2010, 11 (6), 2393–2420. 10.3390/ijms11062393. 20640160 PMC2904924

[ref86] Marrink S. J.; Risselada H. J.; Yefimov S.; Tieleman D. P.; de Vries A. H. The MARTINI Force Field: Coarse Grained Model for Biomolecular Simulations. J. Phys. Chem. B 2007, 111 (27), 7812–7824. 10.1021/jp071097f. 17569554

[ref87] Periole X.; Marrink S.-J. The Martini Coarse-Grained Force Field. In Biomolecular Simulations: Methods and Protocols; Monticelli L., Salonen E., Eds.; Methods in Molecular Biology; Humana Press: Totowa, NJ, 2013; pp 533–565. 10.1007/978-1-62703-017-5_20. 23034762

[ref88] Hoogerbrugge P. J.; Koelman J. M. V. A. Simulating Microscopic Hydrodynamic Phenomena with Dissipative Particle Dynamics. EPL 1992, 19 (3), 155–160. 10.1209/0295-5075/19/3/001.

[ref89] Groot R. D.; Madden T. J. Dynamic Simulation of Diblock Copolymer Microphase Separation. J. Chem. Phys. 1998, 108 (20), 8713–8724. 10.1063/1.476300.

[ref90] Javan Nikkhah S.; Turunen E.; Lepo A.; Ala-Nissila T.; Sammalkorpi M. Multicore Assemblies from Three-Component Linear Homo-Copolymer Systems: A Coarse-Grained Modeling Study. Polymers 2021, 13 (13), 2193 10.3390/polym13132193. 34209428 PMC8272115

[ref91] Harmat A. L.; Javan Nikkhah S.; Sammalkorpi M. Dissipative Particle Dynamics Simulations of H-Shaped Diblock Copolymer Self-Assembly in Solvent. Polymer 2021, 233, 124198 10.1016/j.polymer.2021.124198.

[ref92] Racovita S.; Trofin M.-A.; Loghin D. F.; Zaharia M.-M.; Bucatariu F.; Mihai M.; Vasiliu S. Polybetaines in Biomedical Applications. International Journal of Molecular Sciences 2021, 22 (17), 9321 10.3390/ijms22179321. 34502230 PMC8430529

[ref93] Puri A.; Blumenthal R. Polymeric Lipid Assemblies as Novel Theranostic Tools. Acc. Chem. Res. 2011, 44 (10), 1071–1079. 10.1021/ar2001843. 21919465 PMC3244824

[ref94] Zhang H.; Joubert J. R.; Saavedra S. S. Membranes from Polymerizable Lipids. In Polymer Membranes/Biomembranes; Meier W. P., Knoll W., Eds.; Advances in Polymer Science; Springer: Berlin, Heidelberg, 2010; pp 1–42. 10.1007/978-3-642-10479-4_3.

[ref95] Xuan F.; Liu J. Preparation, Characterization and Application of Zwitterionic Polymers and Membranes: Current Developments and Perspective. Polym. Int. 2009, 58 (12), 1350–1361. 10.1002/pi.2679.

[ref96] Saha P.; Ganguly R.; Li X.; Das R.; Singha N. K.; Pich A. Zwitterionic Nanogels and Microgels: An Overview on Their Synthesis and Applications. Macromol. Rapid Commun. 2021, 42 (13), 2100112 10.1002/marc.202100112. 34021658

[ref97] Jiang S.; Cao Z. Ultralow-Fouling, Functionalizable, and Hydrolyzable Zwitterionic Materials and Their Derivatives for Biological Applications. Adv. Mater. 2010, 22 (9), 920–932. 10.1002/adma.200901407. 20217815

[ref98] Schönemann E.; Laschewsky A.; Wischerhoff E.; Koc J.; Rosenhahn A. Surface Modification by Polyzwitterions of the Sulfabetaine-Type, and Their Resistance to Biofouling. Polymers 2019, 11 (6), 1014 10.3390/polym11061014. PMC6631746 31181764

[ref99] Zhang H.; Chiao M. Anti-Fouling Coatings of Poly(Dimethylsiloxane) Devices for Biological and Biomedical Applications. J. Med. Biol. Eng. 2015, 35 (2), 143–155. 10.1007/s40846-015-0029-4. 25960703 PMC4414934

[ref100] Ilčíková M.; Tkáč J.; Kasák P. Switchable Materials Containing Polyzwitterion Moieties. Polymers 2015, 7 (11), 2344–2370. 10.3390/polym7111518.

[ref101] Paschke S.; Lienkamp K. Polyzwitterions: From Surface Properties and Bioactivity Profiles to Biomedical Applications. ACS Appl. Polym. Mater. 2020, 2 (2), 129–151. 10.1021/acsapm.9b00897.

[ref102] Singh P. K.; Singh V. K.; Singh M. Zwitterionic Polyelectrolytes: A Review. e-Polymers 2007, 7 (1), 030 10.1515/epoly.2007.7.1.335.

[ref103] Neitzel A. E.; De Hoe G. X.; Tirrell M. V. Expanding the Structural Diversity of Polyelectrolyte Complexes and Polyzwitterions. Curr. Opin. Solid State Mater. Sci. 2021, 25 (2), 100897 10.1016/j.cossms.2020.100897.

[ref104] Li M.; Zhuang B.; Yu J. Functional Zwitterionic Polymers on Surface: Structures and Applications. Chem. Asian J. 2020, 15 (14), 2060–2075. 10.1002/asia.202000547. 32506799

[ref105] Biehl P.; Von der Lühe M.; Dutz S.; Schacher F. H. Synthesis, Characterization, and Applications of Magnetic Nanoparticles Featuring Polyzwitterionic Coatings. Polymers 2018, 10 (1), 91 10.3390/polym10010091. PMC6414908 30966126

[ref106] Mah E.; Ghosh R. Thermo-Responsive Hydrogels for Stimuli-Responsive Membranes. Processes 2013, 1 (3), 238–262. 10.3390/pr1030238.

[ref107] de Oliveira O. N. Jr.; Ferreira M.; Da Róz A. L.; de Lima Liete F. Nanostructures; William Andrew, 2016.

[ref108] Sanità G.; Carrese B.; Lamberti A. Nanoparticle Surface Functionalization: How to Improve Biocompatibility and Cellular Internalization. Front. Mol. Biosci. 2020, 7, 587012 10.3389/fmolb.2020.587012. 33324678 PMC7726445

[ref109] Maeda H. Toward a Full Understanding of the EPR Effect in Primary and Metastatic Tumors as Well as Issues Related to Its Heterogeneity. Adv. Drug Delivery Rev. 2015, 91, 3–6. 10.1016/j.addr.2015.01.002. 25579058

[ref110] Muro E.; Pons T.; Lequeux N.; Fragola A.; Sanson N.; Lenkei Z.; Dubertret B. Small and Stable Sulfobetaine Zwitterionic Quantum Dots for Functional Live-Cell Imaging. J. Am. Chem. Soc. 2010, 132 (13), 4556–4557. 10.1021/ja1005493. 20235547

[ref111] Drijvers E.; Liu J.; Harizaj A.; Wiesner U.; Braeckmans K.; Hens Z.; Aubert T. Efficient Endocytosis of Inorganic Nanoparticles with Zwitterionic Surface Functionalization. ACS Appl. Mater. Interfaces 2019, 11 (42), 38475–38482. 10.1021/acsami.9b12398. 31559824

[ref112] Peetla C.; Stine A.; Labhasetwar V. Biophysical Interactions with Model Lipid Membranes: Applications in Drug Discovery and Drug Delivery. Mol. Pharmaceutics 2009, 6 (5), 1264–1276. 10.1021/mp9000662. PMC2757518 19432455

[ref113] Megariotis G.; Romanos N.; Avramopoulos A.; Mikaelian G.; Theodorou D. N. In Silico Study of Levodopa in Hydrated Lipid Bilayers at the Atomistic Level. Journal of Molecular Graphics and Modelling 2021, 107, 107972 10.1016/j.jmgm.2021.107972. 34174554

[ref114] Ji F.; Sun H.; Qin Z.; Zhang E.; Cui J.; Wang J.; Li S.; Yao F. Engineering Polyzwitterion and Polydopamine Decorated Doxorubicin-Loaded Mesoporous Silica Nanoparticles as a PH-Sensitive Drug Delivery. Polymers 2018, 10 (3), 326 10.3390/polym10030326. PMC6415439 30966361

[ref115] Kostova B.; Kamenska E.; Momekov G.; Rachev D.; Georgiev G.; Balashev K. Synthesis and Characterization of Novel Drug Delivery Nanoparticles Based on Polyzwitterionic Copolymers. Eur. Polym. J. 2013, 49 (3), 637–645. 10.1016/j.eurpolymj.2012.12.003.

[ref116] Kostova B.; Kamenska E.; Georgieva D.; Balashev K.; Rachev D.; Georgiev G. Design and Concept of Polyzwitterionic Copolymer Microgel Drug Delivery Systems In Situ Loaded with Non-Steroidal Anti-Inflammatory Ibuprofen. AAPS PharmSciTech 2017, 18 (1), 166–174. 10.1208/s12249-016-0503-5. 26931442

[ref117] Wang J.; Yuan S.; Zhang Y.; Wu W.; Hu Y.; Jiang X. The Effects of Poly(Zwitterions)s versus Poly(Ethylene Glycol) Surface Coatings on the Biodistribution of Protein Nanoparticles. Biomater. Sci. 2016, 4 (9), 1351–1360. 10.1039/C6BM00201C. 27426309

[ref118] Cheng L.; Li Y.; Zhai X.; Xu B.; Cao Z.; Liu W. Polycation-b-Polyzwitterion Copolymer Grafted Luminescent Carbon Dots As a Multifunctional Platform for Serum-Resistant Gene Delivery and Bioimaging. ACS Appl. Mater. Interfaces 2014, 6 (22), 20487–20497. 10.1021/am506076r. 25285670

[ref119] Li Z.; Li H.; Sun Z.; Hao B.; Lee T.-C.; Feng A.; Zhang L.; H. Thang S. Synthesis of Star-Shaped Polyzwitterions with Adjustable UCST and Fast Responsiveness by a Facile RAFT Polymerization. Polym. Chem. 2020, 11 (18), 3162–3168. 10.1039/D0PY00318B.

[ref120] Zhang X.; Chen W.; Zhu X.; Lu Y. Encapsulating Therapeutic Proteins with Polyzwitterions for Lower Macrophage Nonspecific Uptake and Longer Circulation Time. ACS Appl. Mater. Interfaces 2017, 9 (9), 7972–7978. 10.1021/acsami.6b16413. 28194937

[ref121] Chen S.; Zhong Y.; Fan W.; Xiang J.; Wang G.; Zhou Q.; Wang J.; Geng Y.; Sun R.; Zhang Z.; Piao Y.; Wang J.; Zhuo J.; Cong H.; Jiang H.; Ling J.; Li Z.; Yang D.; Yao X.; Xu X.; Zhou Z.; Tang J.; Shen Y. Enhanced Tumour Penetration and Prolonged Circulation in Blood of Polyzwitterion–Drug Conjugates with Cell-Membrane Affinity. Nat. Biomed Eng. 2021, 5 (9), 1019–1037. 10.1038/s41551-021-00701-4. 33859387

[ref122] Li B.; Yuan Z.; He Y.; Hung H.-C.; Jiang S. Zwitterionic Nanoconjugate Enables Safe and Efficient Lymphatic Drug Delivery. Nano Lett. 2020, 20 (6), 4693–4699. 10.1021/acs.nanolett.0c01713. 32379455

[ref123] Zhou B.; Cheng P.; Jiang J.; Chen J.; Zhang L.; Liang J.; Wu W.; Meng Q.; Li J. Engineering Polyzwitterion with Acylsulfonamide-Based Betaine Structure for Protonated Switch of Surface Chemistry at Tumoral PH and Reductive Responsive Drug Release of Polymeric Micelles. Materials Today Chemistry 2020, 17, 100339 10.1016/j.mtchem.2020.100339.

[ref124] Jiang T.; Aseyev V.; Niskanen J.; Hietala S.; Zhang Q.; Tenhu H. Polyzwitterions with LCST Side Chains: Tunable Self-Assembly. Macromolecules 2020, 53 (19), 8267–8275. 10.1021/acs.macromol.0c01708. 33122865 PMC7586405

[ref125] Banskota S.; Yousefpour P.; Kirmani N.; Li X.; Chilkoti A. Long Circulating Genetically Encoded Intrinsically Disordered Zwitterionic Polypeptides for Drug Delivery. Biomaterials 2019, 192, 475–485. 10.1016/j.biomaterials.2018.11.012. 30504081 PMC6376874

[ref126] Nizardo N. M.; Schanzenbach D.; Schönemann E.; Laschewsky A. Exploring Poly(Ethylene Glycol)-Polyzwitterion Diblock Copolymers as Biocompatible Smart Macrosurfactants Featuring UCST-Phase Behavior in Normal Saline Solution. Polymers 2018, 10 (3), 325 10.3390/polym10030325. PMC6414896 30966360

[ref127] Konno T.; Watanabe J.; Ishihara K. Enhanced Solubility of Paclitaxel Using Water-Soluble and Biocompatible 2-Methacryloyloxyethyl Phosphorylcholine Polymers. J. Biomed. Mater. Res., Part A 2003, 65A (2), 209–214. 10.1002/jbm.a.10481. 12734814

[ref128] Iwasaki Y.; Maie H.; Akiyoshi K. Cell-Specific Delivery of Polymeric Nanoparticles to Carbohydrate-Tagging Cells. Biomacromolecules 2007, 8 (10), 3162–3168. 10.1021/bm700606z. 17883278

[ref129] Miyata R.; Ueda M.; Jinno H.; Konno T.; Ishihara K.; Ando N.; Kitagawa Y. Selective Targeting by PreS1 Domain of Hepatitis B Surface Antigen Conjugated with Phosphorylcholine-Based Amphiphilic Block Copolymer Micelles as a Biocompatible, Drug Delivery Carrier for Treatment of Human Hepatocellular Carcinoma with Paclitaxel. Int. J. Cancer 2009, 124 (10), 2460–2467. 10.1002/ijc.24227. 19173297

[ref130] Colley H. E.; Hearnden V.; Avila-Olias M.; Cecchin D.; Canton I.; Madsen J.; MacNeil S.; Warren N.; Hu K.; McKeating J. A.; Armes S. P.; Murdoch C.; Thornhill M. H.; Battaglia G. Polymersome-Mediated Delivery of Combination Anticancer Therapy to Head and Neck Cancer Cells: 2D and 3D in Vitro Evaluation. Mol. Pharmaceutics 2014, 11 (4), 1176–1188. 10.1021/mp400610b. 24533501

[ref131] Massignani M.; LoPresti C.; Blanazs A.; Madsen J.; Armes S. P.; Lewis A. L.; Battaglia G. Controlling Cellular Uptake by Surface Chemistry, Size, and Surface Topology at the Nanoscale. Small 2009, 5 (21), 2424–2432. 10.1002/smll.200900578. 19634187

[ref132] Pegoraro C.; Cecchin D.; Gracia L. S.; Warren N.; Madsen J.; Armes S. P.; Lewis A.; MacNeil S.; Battaglia G. Enhanced Drug Delivery to Melanoma Cells Using PMPC-PDPA Polymersomes. Cancer Letters 2013, 334 (2), 328–337. 10.1016/j.canlet.2013.02.007. 23402813

[ref133] Giacomelli C.; Le Men L.; Borsali R.; Lai-Kee-Him J.; Brisson A.; Armes S. P.; Lewis A. L. Phosphorylcholine-Based PH-Responsive Diblock Copolymer Micelles as Drug Delivery Vehicles: Light Scattering, Electron Microscopy, and Fluorescence Experiments. Biomacromolecules 2006, 7 (3), 817–828. 10.1021/bm0508921. 16529419

[ref134] Du J.; Fan L.; Liu Q. PH-Sensitive Block Copolymer Vesicles with Variable Trigger Points for Drug Delivery. Macromolecules 2012, 45 (20), 8275–8283. 10.1021/ma3015728.

[ref135] Tu S.; Chen Y.-W.; Qiu Y.-B.; Zhu K.; Luo X.-L. Enhancement of Cellular Uptake and Antitumor Efficiencies of Micelles with Phosphorylcholine. Macromol. Biosci. 2011, 11 (10), 1416–1425. 10.1002/mabi.201100111. 21793214

[ref136] Licciardi M.; Craparo E. F.; Giammona G.; Armes S. P.; Tang Y.; Lewis A. L. In Vitro Biological Evaluation of Folate-Functionalized Block Copolymer Micelles for Selective Anti-Cancer Drug Delivery. Macromol. Biosci. 2008, 8 (7), 615–626. 10.1002/mabi.200800009. 18432597

[ref137] Chu H.; Liu N.; Wang X.; Jiao Z.; Chen Z. Morphology and in Vitro Release Kinetics of Drug-Loaded Micelles Based on Well-Defined PMPC–b–PBMA Copolymer. Int. J. Pharm. 2009, 371 (1), 190–196. 10.1016/j.ijpharm.2008.12.033. 19162151

[ref138] Liu G.-Y.; Lv L.-P.; Chen C.-J.; Liu X.-S.; Hu X.-F.; Ji J. Biocompatible and Biodegradable Polymersomes for PH-Triggered Drug Release. Soft Matter 2011, 7 (14), 6629–6636. 10.1039/c1sm05308f.

[ref139] Liu G.-Y.; Lv L.-P.; Chen C.-J.; Hu X.-F.; Ji J. Biocompatible Poly(D,L-Lactide)-Block-Poly(2-Methacryloyloxyethylphosphorylcholine) Micelles for Drug Delivery. Macromol. Chem. Phys. 2011, 212 (6), 643–651. 10.1002/macp.201000735.

[ref140] Liu G.; Jin Q.; Liu X.; Lv L.; Chen C.; Ji J. Biocompatible Vesicles Based on PEO-b-PMPC/α-Cyclodextrin Inclusion Complexes for Drug Delivery. Soft Matter 2011, 7 (2), 662–669. 10.1039/C0SM00708K.

[ref141] Wang H.; Xu F.; Wang Y.; Liu X.; Jin Q.; Ji J. PH-Responsive and Biodegradable Polymeric Micelles Based on Poly(β-Amino Ester)-Graft-Phosphorylcholine for Doxorubicin Delivery. Polym. Chem. 2013, 4 (10), 3012–3019. 10.1039/c3py00139c.

[ref142] Xu J.-P.; Ji J.; Chen W.-D.; Shen J.-C. Novel Biomimetic Polymersomes as Polymer Therapeutics for Drug Delivery. J. Controlled Release 2005, 107 (3), 502–512. 10.1016/j.jconrel.2005.06.013. 16154659

[ref143] Xu J.-P.; Ji J.; Chen W.-D.; Shen J.-C. Novel Biomimetic Surfactant: Synthesis and Micellar Characteristics. Macromol. Biosci. 2005, 5 (2), 164–171. 10.1002/mabi.200400139. 15729722

[ref144] Cao Z.; Yu Q.; Xue H.; Cheng G.; Jiang S. Nanoparticles for Drug Delivery Prepared from Amphiphilic PLGA Zwitterionic Block Copolymers with Sharp Contrast in Polarity between Two Blocks. Angew. Chem., Int. Ed. 2010, 49 (22), 3771–3776. 10.1002/anie.200907079. 20397173

[ref145] Li A.; Luehmann H. P.; Sun G.; Samarajeewa S.; Zou J.; Zhang S.; Zhang F.; Welch M. J.; Liu Y.; Wooley K. L. Synthesis and In Vivo Pharmacokinetic Evaluation of Degradable Shell Cross-Linked Polymer Nanoparticles with Poly(Carboxybetaine) versus Poly(Ethylene Glycol) Surface-Grafted Coatings. ACS Nano 2012, 6 (10), 8970–8982. 10.1021/nn303030t. 23043240 PMC3485677

[ref146] Lin W.; He Y.; Zhang J.; Wang L.; Wang Z.; Ji F.; Chen S. Highly Hemocompatible Zwitterionic Micelles Stabilized by Reversible Cross-Linkage for Anti-Cancer Drug Delivery. Colloids Surf., B 2014, 115, 384–390. 10.1016/j.colsurfb.2013.12.020. 24503292

[ref147] Cao Z.; Zhang L.; Jiang S. Superhydrophilic Zwitterionic Polymers Stabilize Liposomes. Langmuir 2012, 28 (31), 11625–11632. 10.1021/la302433a. 22783927

[ref148] Zhang L.; Xue H.; Cao Z.; Keefe A.; Wang J.; Jiang S. Multifunctional and Degradable Zwitterionic Nanogels for Targeted Delivery, Enhanced MR Imaging, Reduction-Sensitive Drug Release, and Renal Clearance. Biomaterials 2011, 32 (20), 4604–4608. 10.1016/j.biomaterials.2011.02.064. 21453965

[ref149] Cheng G.; Mi L.; Cao Z.; Xue H.; Yu Q.; Carr L.; Jiang S. Functionalizable and Ultrastable Zwitterionic Nanogels. Langmuir 2010, 26 (10), 6883–6886. 10.1021/la100664g. 20405859

[ref150] Soma D.; Kitayama J.; Konno T.; Ishihara K.; Yamada J.; Kamei T.; Ishigami H.; Kaisaki S.; Nagawa H. Intraperitoneal Administration of Paclitaxel Solubilized with Poly(2-Methacryloxyethyl Phosphorylcholine-Co n-Butyl Methacrylate) for Peritoneal Dissemination of Gastric Cancer. Cancer Science 2009, 100 (10), 1979–1985. 10.1111/j.1349-7006.2009.01265.x. 19604244 PMC11159799

[ref151] Gooneie A.; Schuschnigg S.; Holzer C. A Review of Multiscale Computational Methods in Polymeric Materials. Polymers 2017, 9 (1), 16 10.3390/polym9010016. PMC6432151 30970697

[ref152] Shao Q.; Jiang S. Effect of Carbon Spacer Length on Zwitterionic Carboxybetaines. J. Phys. Chem. B 2013, 117 (5), 1357–1366. 10.1021/jp3094534. 23316760

[ref153] Cong W.; Liu Q.; Liang Q.; Wang Y.; Luo G. Investigation on the Interactions between Pirarubicin and Phospholipids. Biophys. Chem. 2009, 143 (3), 154–160. 10.1016/j.bpc.2009.05.005. 19481330

[ref154] Metropolis N.; Rosenbluth A. W.; Rosenbluth M. N.; Teller A. H.; Teller E. Equation of State Calculations by Fast Computing Machines. J. Chem. Phys. 1953, 21 (6), 1087–1092. 10.1063/1.1699114. 41342507

[ref155] Chen Y.; Schultz A. J.; Errington J. R. Coupled Monte Carlo and Molecular Dynamics Simulations on Interfacial Properties of Antifouling Polymer Membranes. J. Phys. Chem. B 2021, 125 (29), 8193–8204. 10.1021/acs.jpcb.1c01966. 34259529

[ref156] Li C.; Li M.; Qi W.; Su R.; Yu J. Effect of Hydrophobicity and Charge Separation on the Antifouling Properties of Surface-Tethered Zwitterionic Peptides. Langmuir 2021, 37 (28), 8455–8462. 10.1021/acs.langmuir.1c00803. 34228454

[ref157] Teng J.; Liu Y.; Shen Z.; Lv W.; Chen Y. Molecular Simulation of Zwitterionic Polypeptides on Protecting Glucagon-like Peptide-1 (GLP-1). Int. J. Biol. Macromol. 2021, 174, 519–526. 10.1016/j.ijbiomac.2021.01.207. 33539961

[ref158] Man V. H.; Li M. S.; Derreumaux P.; Wang J.; Nguyen P. H. Molecular Mechanism of Ultrasound-Induced Structural Defects in Liposomes: A Nonequilibrium Molecular Dynamics Simulation Study. Langmuir 2021, 37 (26), 7945–7954. 10.1021/acs.langmuir.1c00555. 34161100

[ref159] Cooke I. R.; Kremer K.; Deserno M. Tunable Generic Model for Fluid Bilayer Membranes. Phys. Rev. E 2005, 72 (1), 011506 10.1103/PhysRevE.72.011506. 16089969

[ref160] Quan X.; Zhao D.; Li L.; Zhou J. Understanding the Cellular Uptake of PH-Responsive Zwitterionic Gold Nanoparticles: A Computer Simulation Study. Langmuir 2017, 33 (50), 14480–14489. 10.1021/acs.langmuir.7b03544. 29166558

[ref161] Das M.; Dahal U.; Mesele O.; Liang D.; Cui Q. Molecular Dynamics Simulation of Interaction between Functionalized Nanoparticles with Lipid Membranes: Analysis of Coarse-Grained Models. J. Phys. Chem. B 2019, 123 (49), 10547–10561. 10.1021/acs.jpcb.9b08259. 31675790

[ref162] Zeng Q. H.; Yu A. B.; Lu G. Q. Multiscale Modeling and Simulation of Polymer Nanocomposites. Prog. Polym. Sci. 2008, 33 (2), 191–269. 10.1016/j.progpolymsci.2007.09.002.

[ref163] Groot R. D. Electrostatic Interactions in Dissipative Particle Dynamics—Simulation of Polyelectrolytes and Anionic Surfactants. J. Chem. Phys. 2003, 118 (24), 11265–11277. 10.1063/1.1574800.

[ref164] Espanol P. Dissipative Particle Dynamics. In Handbook of Materials Modeling: Methods; Yip S., Ed.; Springer Netherlands: Dordrecht, 2005; pp 2503–2512. 10.1007/978-1-4020-3286-8_131.

[ref165] Zeng S.; Quan X.; Zhu H.; Sun D.; Miao Z.; Zhang L.; Zhou J. Computer Simulations on a PH-Responsive Anticancer Drug Delivery System Using Zwitterion-Grafted Polyamidoamine Dendrimer Unimolecular Micelles. Langmuir 2021, 37 (3), 1225–1234. 10.1021/acs.langmuir.0c03217. 33417464

[ref166] Min W.; Zhao D.; Quan X.; Sun D.; Li L.; Zhou J. Computer Simulations on the PH-Sensitive Tri-Block Copolymer Containing Zwitterionic Sulfobetaine as a Novel Anti-Cancer Drug Carrier. Colloids Surf., B 2017, 152, 260–268. 10.1016/j.colsurfb.2017.01.033. 28119221

[ref167] Laschewsky A.; Rosenhahn A. Molecular Design of Zwitterionic Polymer Interfaces: Searching for the Difference. Langmuir 2019, 35 (5), 1056–1071. 10.1021/acs.langmuir.8b01789. 30048142

[ref168] Piatkovsky M.; Acar H.; Marciel A. B.; Tirrell M.; Herzberg M. A Zwitterionic Block-Copolymer, Based on Glutamic Acid and Lysine, Reduces the Biofouling of UF and RO Membranes. J. Membr. Sci. 2018, 549, 507–514. 10.1016/j.memsci.2017.12.042.

[ref169] Kane R. S.; Deschatelets P.; Whitesides G. M. Kosmotropes Form the Basis of Protein-Resistant Surfaces. Langmuir 2003, 19 (6), 2388–2391. 10.1021/la020737x.

[ref170] Encinas N.; Angulo M.; Astorga C.; Colilla M.; Izquierdo-Barba I.; Vallet-Regí M. Mixed-Charge Pseudo-Zwitterionic Mesoporous Silica Nanoparticles with Low-Fouling and Reduced Cell Uptake Properties. Acta Biomaterialia 2019, 84, 317–327. 10.1016/j.actbio.2018.12.012. 30529082 PMC6718287

[ref171] Venault A.; Wei T.-C.; Shih H.-L.; Yeh C.-C.; Chinnathambi A.; Alharbi S. A.; Carretier S.; Aimar P.; Lai J.-Y.; Chang Y. Antifouling Pseudo-Zwitterionic Poly(Vinylidene Fluoride) Membranes with Efficient Mixed-Charge Surface Grafting via Glow Dielectric Barrier Discharge Plasma-Induced Copolymerization. J. Membr. Sci. 2016, 516, 13–25. 10.1016/j.memsci.2016.05.044.

[ref172] Liu X.; Jin Q.; Ji Y.; Ji J. Minimizing Nonspecific Phagocytic Uptake of Biocompatible Gold Nanoparticles with Mixed Charged Zwitterionic Surface Modification. J. Mater. Chem. 2012, 22 (5), 1916–1927. 10.1039/C1JM14178C.

[ref173] Fujii S.; Kido M.; Sato M.; Higaki Y.; Hirai T.; Ohta N.; Kojio K.; Takahara A. PH-Responsive and Selective Protein Adsorption on an Amino Acid-Based Zwitterionic Polymer Surface. Polym. Chem. 2015, 6 (39), 7053–7059. 10.1039/C5PY00783F.

[ref174] Liu Q.; Li W.; Wang H.; Newby B. Z.; Cheng F.; Liu L. Amino Acid-Based Zwitterionic Polymer Surfaces Highly Resist Long-Term Bacterial Adhesion. Langmuir 2016, 32 (31), 7866–7874. 10.1021/acs.langmuir.6b01329. 27397718

[ref175] Koeberle P.; Laschewsky A. Hydrophobically Modified Zwitterionic Polymers: Synthesis, Bulk Properties, and Miscibility with Inorganic Salts. Macromolecules 1994, 27 (8), 2165–2173. 10.1021/ma00086a028.

[ref176] Anton P.; Laschewsky A. Solubilization by Polysoaps. Colloid Polym. Sci. 1994, 272 (9), 1118–1128. 10.1007/BF00652381.

[ref177] Bonte N.; Laschewsky A. Zwitterionic Polymers with Carbobetaine Moieties. Polymer 1996, 37 (10), 2011–2019. 10.1016/0032-3861(96)87319-8.

[ref178] Grassl B.; Francois J.; Billon L. Associating Behaviour of Polyacrylamide Modified with a New Hydrophobic Zwitterionic Monomer. Polym. Int. 2001, 50 (10), 1162–1169. 10.1002/pi.751.

[ref179] Kratz K.; Breitenkamp K.; Hule R.; Pochan D.; Emrick T. PC-Polyolefins: Synthesis and Assembly Behavior in Water. Macromolecules 2009, 42 (9), 3227–3229. 10.1021/ma900653h.

[ref180] Letchford K.; Burt H. A Review of the Formation and Classification of Amphiphilic Block Copolymer Nanoparticulate Structures: Micelles, Nanospheres, Nanocapsules and Polymersomes. Eur. J. Pharm. Biopharm 2007, 65 (3), 259–269. 10.1016/j.ejpb.2006.11.009. 17196803

[ref181] Antonietti M.; Förster S. Vesicles and Liposomes: A Self-Assembly Principle Beyond Lipids. Adv. Mater. 2003, 15 (16), 1323–1333. 10.1002/adma.200300010.

[ref182] Marsden H. R.; Gabrielli L.; Kros A. Rapid Preparation of Polymersomes by a Water Addition/Solvent Evaporation Method. Polym. Chem. 2010, 1 (9), 1512–1518. 10.1039/c0py00172d.

[ref183] Harada A.; Matsuki R.; Ichimura S.; Yuba E.; Kono K. Intracellular Environment-Responsive Stabilization of Polymer Vesicles Formed from Head-Tail Type Polycations Composed of a Polyamidoamine Dendron and Poly(L-Lysine). Molecules 2013, 18 (10), 12168–12179. 10.3390/molecules181012168. 24084020 PMC6269863

[ref184] Lin Y.-L.; Chang H.-Y.; Sheng Y.-J.; Tsao H.-K. The Fusion Mechanism of Small Polymersomes Formed by Rod–Coil Diblock Copolymers. Soft Matter 2014, 10 (10), 1500–1511. 10.1039/c3sm52387j. 24652278

[ref185] Shinoda W.; DeVane R.; Klein M. L. Zwitterionic Lipid Assemblies: Molecular Dynamics Studies of Monolayers, Bilayers, and Vesicles Using a New Coarse Grain Force Field. J. Phys. Chem. B 2010, 114 (20), 6836–6849. 10.1021/jp9107206. 20438090 PMC2876730

[ref186] De Leo V.; Milano F.; Agostiano A.; Catucci L. Recent Advancements in Polymer/Liposome Assembly for Drug Delivery: From Surface Modifications to Hybrid Vesicles. Polymers 2021, 13 (7), 1027 10.3390/polym13071027. 33810273 PMC8037206

[ref187] Di J.; Xie F.; Xu Y. When Liposomes Met Antibodies: Drug Delivery and Beyond. Adv. Drug Delivery Rev. 2020, 154–155, 151–162. 10.1016/j.addr.2020.09.003. 32926944

[ref188] Liao M.; Liu H.; Guo H.; Zhou J. Mesoscopic Structures of Poly(Carboxybetaine) Block Copolymer and Poly(Ethylene Glycol) Block Copolymer in Solutions. Langmuir 2017, 33 (30), 7575–7582. 10.1021/acs.langmuir.7b01610. 28689413

[ref189] Groot R. D. Mesoscopic Simulation of Polymer–Surfactant Aggregation. Langmuir 2000, 16 (19), 7493–7502. 10.1021/la000010d.

[ref190] Abbasi E.; Aval S. F.; Akbarzadeh A.; Milani M.; Nasrabadi H. T.; Joo S. W.; Hanifehpour Y.; Nejati-Koshki K.; Pashaei-Asl R. Dendrimers: Synthesis, Applications, and Properties. Nanoscale Res. Lett. 2014, 9 (1), 247 10.1186/1556-276X-9-247. 24994950 PMC4074873

[ref191] Newkome G. R.; Shreiner C. D. Poly(Amidoamine), Polypropylenimine, and Related Dendrimers and Dendrons Possessing Different 1→2 Branching Motifs: An Overview of the Divergent Procedures. Polymer 2008, 49 (1), 1–173. 10.1016/j.polymer.2007.10.021.

[ref192] Hu J.; Su Y.; Zhang H.; Xu T.; Cheng Y. Design of Interior-Functionalized Fully Acetylated Dendrimers for Anticancer Drug Delivery. Biomaterials 2011, 32 (36), 9950–9959. 10.1016/j.biomaterials.2011.09.016. 21944725

[ref193] Xiong Z.; Wang Y.; Zhu J.; Li X.; He Y.; Qu J.; Shen M.; Xia J.; Shi X. Dendrimers Meet Zwitterions: Development of a Unique Antifouling Nanoplatform for Enhanced Blood Pool, Lymph Node and Tumor CT Imaging. Nanoscale 2017, 9 (34), 12295–12301. 10.1039/C7NR03940A. 28819657

[ref194] Svenningsen S. W.; Janaszewska A.; Ficker M.; Petersen J. F.; Klajnert-Maculewicz B.; Christensen J. B. Two for the Price of One: PAMAM-Dendrimers with Mixed Phosphoryl Choline and Oligomeric Poly(Caprolactone) Surfaces. Bioconjugate Chem. 2016, 27 (6), 1547–1557. 10.1021/acs.bioconjchem.6b00213. 27244598

[ref195] Han Y.; Qian Y.; Zhou X.; Hu H.; Liu X.; Zhou Z.; Tang J.; Shen Y. Facile Synthesis of Zwitterionic Polyglycerol Dendrimers with a β-Cyclodextrin Core as MRI Contrast Agent Carriers. Polym. Chem. 2016, 7 (41), 6354–6362. 10.1039/C6PY01404F.

[ref196] Huang D.; Yang F.; Wang X.; Shen H.; You Y.; Wu D. Facile Synthesis and Self-Assembly Behaviour of PH-Responsive Degradable Polyacetal Dendrimers. Polym. Chem. 2016, 7 (40), 6154–6158. 10.1039/C6PY01511E.

[ref197] Hao L.; Lin L.; Zhou J. PH-Responsive Zwitterionic Copolymer DHA–PBLG–PCB for Targeted Drug Delivery: A Computer Simulation Study. Langmuir 2019, 35 (5), 1944–1953. 10.1021/acs.langmuir.8b00626. 29692174

[ref198] Rai R.; Alwani S.; Badea I. Polymeric Nanoparticles in Gene Therapy: New Avenues of Design and Optimization for Delivery Applications. Polymers (Basel) 2019, 11 (4), 745 10.3390/polym11040745. PMC6523186 31027272

[ref199] Warriner L. W.; Duke J. R.; Pack D. W.; DeRouchey J. E. Succinylated Polyethylenimine Derivatives Greatly Enhance Polyplex Serum Stability and Gene Delivery In Vitro. Biomacromolecules 2018, 19 (11), 4348–4357. 10.1021/acs.biomac.8b01248. 30354068

[ref200] Ghobadi A. F.; Letteri R.; Parelkar S. S.; Zhao Y.; Chan-Seng D.; Emrick T.; Jayaraman A. Dispersing Zwitterions into Comb Polymers for Nonviral Transfection: Experiments and Molecular Simulation. Biomacromolecules 2016, 17 (2), 546–557. 10.1021/acs.biomac.5b01462. 26741292

[ref201] Chen Q.; Ding H.; Zhou J.; Zhao X.; Zhang J.; Yang C.; Li K.; Qiao M.; Hu H.; Ding P.; Zhao X. Novel Glycyrrhetinic Acid Conjugated PH-Sensitive Liposomes for the Delivery of Doxorubicin and Its Antitumor Activities. RSC Adv. 2016, 6 (22), 17782–17791. 10.1039/C6RA01580H.

[ref202] Hao W.; Liu D.; Shang Y.; Zhang J.; Xu S.; Liu H. PH-Triggered Copolymer Micelles as Drug Nanocarriers for Intracellular Delivery. RSC Adv. 2016, 6 (35), 29149–29158. 10.1039/C6RA00673F.

[ref203] Kesharwani P.; Jain K.; Jain N. K. Dendrimer as Nanocarrier for Drug Delivery. Prog. Polym. Sci. 2014, 39 (2), 268–307. 10.1016/j.progpolymsci.2013.07.005.

[ref204] Li J.; Mooney D. J. Designing Hydrogels for Controlled Drug Delivery. Nat. Rev. Mater. 2016, 1 (12), 1–17. 10.1038/natrevmats.2016.71. PMC5898614 29657852

[ref205] Yildiz I.; Shukla S.; Steinmetz N. F. Applications of Viral Nanoparticles in Medicine. Curr. Opin. Biotechnol. 2011, 22 (6), 901–908. 10.1016/j.copbio.2011.04.020. 21592772 PMC3206135

[ref206] Fam S. Y.; Chee C. F.; Yong C. Y.; Ho K. L.; Mariatulqabtiah A. R.; Tan W. S. Stealth Coating of Nanoparticles in Drug-Delivery Systems. Nanomaterials (Basel) 2020, 10 (4), E787 10.3390/nano10040787. 32325941 PMC7221919

[ref207] Wu T.-T.; Zhou S.-H. Nanoparticle-Based Targeted Therapeutics in Head-And-Neck Cancer. Int. J. Med. Sci. 2015, 12 (2), 187–200. 10.7150/ijms.10083. 25589895 PMC4293184

[ref208] Dormont F.; Varna M.; Couvreur P. Nanoplumbers: Biomaterials to Fight Cardiovascular Diseases. Mater. Today 2018, 21 (2), 122–143. 10.1016/j.mattod.2017.07.008.

[ref209] Yang W.; Chen S.; Cheng G.; Vaisocherová H.; Xue H.; Li W.; Zhang J.; Jiang S. Film Thickness Dependence of Protein Adsorption from Blood Serum and Plasma onto Poly(Sulfobetaine)-Grafted Surfaces. Langmuir 2008, 24 (17), 9211–9214. 10.1021/la801487f. 18672924

[ref210] Zhang Z.; Chao T.; Chen S.; Jiang S. Superlow Fouling Sulfobetaine and Carboxybetaine Polymers on Glass Slides. Langmuir 2006, 22 (24), 10072–10077. 10.1021/la062175d. 17107002

[ref211] Ratner B. D.; Hoffman A. S.; Schoen F. J.; Lemons J. E. Biomaterials Science: An Introduction to Materials in Medicine; Elsevier, 2004.

[ref212] Gaberc-Porekar V.; Zore I.; Podobnik B.; Menart V. Obstacles and Pitfalls in PEGylation of Therapeutic Proteins. Curr. Opin. Drug Discovery Dev. 2008, 11, 242–250. 18283612

[ref213] Chang Y.; Chang W.-J.; Shih Y.-J.; Wei T.-C.; Hsiue G.-H. Zwitterionic Sulfobetaine-Grafted Poly(Vinylidene Fluoride) Membrane with Highly Effective Blood Compatibility via Atmospheric Plasma-Induced Surface Copolymerization. ACS Appl. Mater. Interfaces 2011, 3 (4), 1228–1237. 10.1021/am200055k. 21388227

[ref214] Yang W. J.; Neoh K.-G.; Kang E.-T.; Teo S. L.-M.; Rittschof D. Polymer Brush Coatings for Combating Marine Biofouling. Prog. Polym. Sci. 2014, 39 (5), 1017–1042. 10.1016/j.progpolymsci.2014.02.002.

[ref215] Koc J.; Schönemann E.; Amuthalingam A.; Clarke J.; Finlay J. A.; Clare A. S.; Laschewsky A.; Rosenhahn A. Low-Fouling Thin Hydrogel Coatings Made of Photo-Cross-Linked Polyzwitterions. Langmuir 2019, 35 (5), 1552–1562. 10.1021/acs.langmuir.8b02799. 30376714

[ref216] Ma M.-Q.; Zhang C.; Chen T.-T.; Yang J.; Wang J.-J.; Ji J.; Xu Z.-K. Bioinspired Polydopamine/Polyzwitterion Coatings for Underwater Anti-Oil and -Freezing Surfaces. Langmuir 2019, 35 (5), 1895–1901. 10.1021/acs.langmuir.8b02320. 30145900

[ref217] Sanchez-Cano C.; Carril M. Recent Developments in the Design of Non-Biofouling Coatings for Nanoparticles and Surfaces. International Journal of Molecular Sciences 2020, 21 (3), 1007 10.3390/ijms21031007. PMC7037411 32028729

[ref218] Kovacevic M.; Balaz I.; Marson D.; Laurini E.; Jovic B. Mixed-Monolayer Functionalized Gold Nanoparticles for Cancer Treatment: Atomistic Molecular Dynamics Simulations Study. Biosystems 2021, 202, 104354 10.1016/j.biosystems.2021.104354. 33444701

[ref219] Shan Y.; Kim E. T.; Eastwood M. P.; Dror R. O.; Seeliger M. A.; Shaw D. E. How Does a Drug Molecule Find Its Target Binding Site?. J. Am. Chem. Soc. 2011, 133 (24), 9181–9183. 10.1021/ja202726y. 21545110 PMC3221467

[ref220] Huo J.; Chen Z.; Zhou J. Zwitterionic Membrane via Nonsolvent Induced Phase Separation: A Computer Simulation Study. Langmuir 2019, 35 (5), 1973–1983. 10.1021/acs.langmuir.8b01786. 30056719

[ref221] Ishihara K.; Nomura H.; Mihara T.; Kurita K.; Iwasaki Y.; Nakabayashi N. Why Do Phospholipid Polymers Reduce Protein Adsorption?. Journal of Biomedical Materials Research: An Official Journal of The Society for Biomaterials, The Japanese Society for Biomaterials, and the Australian Society for Biomaterials 1998, 39 (2), 323–330. 10.1002/(SICI)1097-4636(199802)39:2<323::AID-JBM21>3.0.CO;2-C. 9457564

[ref222] Vermette P.; Meagher L. Interactions of Phospholipid- and Poly(Ethylene Glycol)-Modified Surfaces with Biological Systems: Relation to Physico-Chemical Properties and Mechanisms. Colloids Surf., B 2003, 28 (2), 153–198. 10.1016/S0927-7765(02)00160-1.

[ref223] Chen S.; Zheng J.; Li L.; Jiang S. Strong Resistance of Phosphorylcholine Self-Assembled Monolayers to Protein Adsorption: Insights into Nonfouling Properties of Zwitterionic Materials. J. Am. Chem. Soc. 2005, 127 (41), 14473–14478. 10.1021/ja054169u. 16218643

[ref224] Chen S.; Liu L.; Jiang S. Strong Resistance of Oligo(Phosphorylcholine) Self-Assembled Monolayers to Protein Adsorption. Langmuir 2006, 22 (6), 2418–2421. 10.1021/la052851w. 16519431

[ref225] Shao Q.; He Y.; White A. D.; Jiang S. Different Effects of Zwitterion and Ethylene Glycol on Proteins. J. Chem. Phys. 2012, 136 (22), 225101 10.1063/1.4726135. 22713073

[ref226] Shao Q.; Jiang S. Influence of Charged Groups on the Properties of Zwitterionic Moieties: A Molecular Simulation Study. J. Phys. Chem. B 2014, 118 (27), 7630–7637. 10.1021/jp5027114. 24922061

[ref227] Fahn S. Levodopa in the Treatment of Parkinson’s Disease. J. Neural Transm Suppl 2006, (71), 1–15. 10.1007/978-3-211-33328-0_1. 17447410

[ref228] Scalise M.; Galluccio M.; Console L.; Pochini L.; Indiveri C. The Human SLC7A5 (LAT1): The Intriguing Histidine/Large Neutral Amino Acid Transporter and Its Relevance to Human Health. Front. Chem. 2018, 6, 243 10.3389/fchem.2018.00243. 29988369 PMC6023973

